# Mapping Aboriginal Mental Health Journeys Through Psychiatric Care Systems

**DOI:** 10.1001/jamanetworkopen.2026.13904

**Published:** 2026-05-20

**Authors:** Helen Milroy, Blerida Banushi, Beatriz Cuesta Briand, Dathan Tran, Shraddha Kashyap, Michael Mitchell, Gray Josephine, Toby Price, Michael Taran, Layale Tayba, Mathew Coleman, Jemma Collova, Sean Hood, Patricia Dudgeon, Michael Small

**Affiliations:** 1 UWA Medical School, The University of Western Australia, Perth, Western Australia, Australia; 2 The Kids Research Institute Australia, Nedlands, Western Australia, Australia; 3 School of Indigenous Studies, The University of Western Australia, Crawley, Western Australia, Australia; 4 Rural Clinical School of WA, The University of Western Australia, Crawley, Western Australia, Australia; 5 Complex Systems Group, Department of Mathematics and Statistics, The University of Western Australia, Crawley, Western Australia, Australia; 6 WA Country Health Service, Perth, Western Australia, Australia; 7 Great Southern Mental Health Service, WA Country Health Service, Albany, Western Australia, Australia; 8 Rural Clinical School of WA, The University of Western Australia, Crawley, Western Australia, Australia; 9 Division of Psychiatry, UWA Medical School, The University of Western Australia, Crawley, Western Australia, Australia

## Abstract

**Importance:**

Mental health services fail to provide culturally safe care for Aboriginal peoples, contributing to persistent health disparities. Understanding how psychiatric care is organized, alongside lived experiences of care, may identify leverage points for service redesign.

**Objective:**

To examine Aboriginal mental health care pathways using integrated qualitative and quantitative analysis.

**Design, Setting, and Participants:**

This mixed-methods study was conducted from June 2022 to December 2023 at the Great Southern Mental Health Service in Western Australia. Twenty Aboriginal adults were recruited; the quantitative component included 19 patients with 1108 documented clinical interactions, and 7 participated in in-depth yarning interviews. Novel analytical methods included a community-preserving surrogate network algorithm and transition-matrix trajectory clustering.

**Main Outcomes and Measures:**

Centrality metrics identifying key agents in the clinical interaction network; patient trajectory clustering based on clinical action sequences; qualitative themes from yarning interviews using reflexive thematic analysis.

**Results:**

A total of 20 Aboriginal adults were recruited; the quantitative analysis included 19 patients (mean [SD] age, 38.4 [15.9] years; 10 women and 9 men) with 1108 documented interactions, with 7 patients (mean [SD] age, 44.0 [17.8] years; 4 women and 3 men) participating in qualitative yarning interviews. The clinical interaction network had a core-periphery structure in which patients often served as the main link between external agents and hospital staff. Aboriginal mental health workers had closeness centrality above the 99th percentile in surrogate networks, indicating a structurally distinctive central position within the network. Trajectory analysis identified 3 care pathways: predominantly internal care, prolonged internal engagement, and complex external referral with repeated readmissions. Qualitative themes emphasized cultural safety, kinship, and ongoing trauma and showed inconsistent Aboriginal mental health worker presence during crises, while integrated analyses highlighted their pivotal yet vulnerable role within fragmented care pathways, underscoring the need for trauma-informed, culturally safe redesign.

**Conclusions and Relevance:**

In this mixed-methods study, Aboriginal mental health workers occupied central bridging positions, and patients were intermediaries between disconnected parts of the system. Strengthening culturally grounded roles and redesigning care pathways based on operational information flow may improve care coordination and cultural safety in multisetting mental health systems.

## Key Points

**Question:**

How are Aboriginal mental health care pathways structured, and how do network structure, care coordination, and experiences of cultural safety indicate where services should be redesigned?

**Findings:**

In this mixed-methods study of 20 Aboriginal patients, Aboriginal mental health workers occupied a unique central position in the care network, while patients served as connectors between fragmented services and followed nonlinear care pathways. Qualitative findings emphasized cultural safety, kinship, and trauma-informed care and highlighted inconsistent Aboriginal mental health worker presence.

**Meaning:**

These results suggest that embedding culturally grounded roles and redesigning care pathways may improve coordination and cultural safety in fragmented health systems.

## Teaser

This mixed-methods study including Aboriginal patients in an Australian mental health system examines key overlaps and gaps in culturally sensitive care and associated services.

## Introduction

Aboriginal and Torres Strait Islander peoples, the First Peoples of Australia, possess rich cultural traditions and holistic concepts of health that have sustained well-being for millennia. However, mainstream psychiatric services grounded in Western biomedical paradigms overlook Indigenous understandings of health and healing.^1^ This oversight reflects limitations of the biopsychosocial model which, like the biomedical approach, fails to incorporate Aboriginal health concepts and perpetuates culturally unsafe care environments and ongoing disparities in mental health outcomes between Indigenous and non-Indigenous populations.^1,2,3^

Cultural safety in health care refers to care that enhances the collective empowerment of Aboriginal peoples by taking into account Aboriginal concepts of health and well-being.^4^ It goes beyond cultural awareness or sensitivity to examine power imbalances, institutional discrimination, and the impact of colonization on health outcomes.^4,5^ For Aboriginal peoples, well-being is understood holistically as social and emotional well-being (SEWB) framework, which recognizes the interconnected importance of culture, country, spirituality, ancestry, family, and community to mental health and well-being.^6^ However, many Aboriginal peoples report that mental health services are unwelcoming and alienating, with overt and systemic racism shaping their experiences within the health care system.^1,7,8,9,10,11^ This perception contributes to avoidance of health services, with cultural disconnection and poor communication frequently reported as key drivers of negative experiences.^3,12,13^ Cultural safety is particularly critical in regional settings where geographic isolation and service limitations can exacerbate barriers to effective and culturally safe mental health care for Aboriginal peoples.

We conducted a mixed-methods study at a regional mental health service in Western Australia to examine Aboriginal mental health care pathways through integrated lived experience and network analysis. Guided by Aboriginal leadership through the Aboriginal Participatory Action Research (APAR) framework,^14^ this study identifies structural dependencies, information flow patterns, and cultural safety experiences to inform culturally safe, community-led service redesign.

## Methods

### Study Design and Participants

We conducted a convergent mixed-methods study at the Great Southern Mental Health Service (GSMHS), the regional public mental health provider in Western Australia, to examine Aboriginal mental health patient journeys. This mental health service delivers integrated acute psychiatric assessment, treatment, and ongoing care spanning emergency, inpatient, and outpatient settings.

We recruited 20 Aboriginal adults who had accessed GSMHS in the preceding 12 months. Aboriginal status was self-identified at recruitment and was assessed because the study focused on mental health care pathways and cultural safety experiences among Aboriginal peoples. Seven participated in qualitative interviews and 19 in the quantitative group; 6 individuals contributed to both components. Reporting followed the Consolidated Criteria for Reporting Qualitative Research (COREQ) reporting guideline for the qualitative component and the Good Reporting of a Mixed Methods Study (GRAMMS)^15^ guideline for mixed-methods reporting and integration (eTable 2 in Supplement 1). The study was also guided by the APAR framework^14^ and principles from the Aboriginal and Torres Strait Islander Quality Appraisal Tool^16^ (eTable 3 in Supplement 1) to ensure Indigenous methodological integrity, Aboriginal leadership, community partnership, and cultural relevance in study design, data collection, interpretation, and reporting. Aboriginal researchers led the study in partnership with community organizations through an iterative co-design process. Ethical approval was granted by the Western Australian Aboriginal Health Ethics Committee and the Research Governance Service; all participants provided written informed consent.

### Network Data Collection

Clinical staff recorded detailed event logs of interactions involving 19 Aboriginal patients admitted at GSMHS between June 2022 and December 2023. These logs captured agents involved, information systems accessed, actions taken, and outcomes, yielding 1108 interaction instances (eMethods in Supplement 1). Agents referred to the people or role-based participants involved in each clinical interaction, including the patient, clinical staff, family members, and external services.

### Sharing Lived Experiences (Qualitative Data Collection)

Qualitative data were collected via research yarning,^18^ an Indigenous qualitative method that uses culturally grounded, relational conversation to support respectful, participant-led sharing of experiences, and were facilitated by 2 Aboriginal researchers in accordance with culturally appropriate methods (detailed in eTable 3 in Supplement 1). All interviews were audio-recorded, transcribed and deidentified. The yarning guide was codeveloped with the Aboriginal governance team to explore cultural safety experiences with mental health services (eTable 4 in Supplement 1). Participant well-being was prioritized throughout.

### Data Analysis

To examine how care was organized across service, we used the interaction logs to construct an undirected clinical interaction network (CIN), following the approach of McCullough et al,^17^ in which nodes represented agents and information systems. Edges connected nodes that appeared in the same recorded interaction; links to information systems were limited to staff with appropriate access so that the network represented feasible care processes rather than all theoretically possible connections (eMethods in Supplement 1). Because interactions varied in frequency, we examined both unweighted and weighted versions of the network. For weighted analyses, distances between connected nodes were defined as the reciprocals of interaction frequency, such that more frequently observed interactions corresponded to shorter path lengths.

We calculated degree, closeness, and betweenness centrality to identify roles that were highly connected (degree), centrally located (closeness), or bridging otherwise separate parts of care (betweenness). To test whether high centrality reflected a role’s structural position rather than number of connections alone, we performed surrogate network testing using closeness centrality on the unweighted CIN (eMethods in Supplement 1). Standard double-edge swap (DES) randomization preserved the number of connections for each node, but disrupted the clinically relevant structure of the network, particularly the disconnect between external agents and hospital staff, and was therefore not an appropriate null model (eMethods in Supplement 1). We therefore developed a community-preserving DES (CP-DES) algorithm that preserved node degree and the number of connections within and between organizational groups (external agents or hospital staff) while randomizing local network structure (eMethods in Supplement 1). This allowed us to assess whether a role’s observed closeness centrality was greater than expected based on its number of connections and broader service structure.

To examine individual care pathways, we grouped the recorded interaction and outcome entries from each event log into 13 standardized action categories and ordered them chronologically for each patient as a sequence of care events (eMethods in Supplement 1). These categories captured the main types of clinical activity, such as assessment, intervention, referral, and disposition, allowing pathways to be compared across patients using a common framework. Because exact time intervals between events were not available, we focused on the order of care actions and the transitions from one action to the next. We summarized the diversity of actions within each patient’s sequence using Shannon entropy and constructed patient-specific transition matrices to quantify how often each action was followed by another (eMethods in Supplement 1). We then compared patients’ transition matrices using Jaccard distance to measure similarity between care trajectories and used these distances to build a patient similarity network with ε-nearest-neighbor thresholding at ε = 0.8. Greedy modularity maximization was applied to this network to identify groups of patients with similar clinical pathways (eMethods in Supplement 1).

Qualitative reporting followed the COREQ reporting guideline. Details on interviewer positioning, recruitment and consent, interview procedures, analytic approach, team-based reflexive interpretation, and participant and community feedback are available in eTable 2 and eMethods in Supplement 1.

Results from mixed-methods analysis were reported in accordance with GRAMMS^15^ (eTable 2 in Supplement 1). Qualitative and quantitative findings were analyzed independently and integrated at the interpretation stage through side-by-side comparison in a joint display, with meta-inferences refined through joint sense-making by Aboriginal and quantitative researchers. Because 6 participants contributed to both study components, integration focused on patterns across datasets rather than full participant-level matching. Qualitative findings were used to contextualize and interpret the quantitative results, while the SEWB framework^6^ provided a culturally grounded lens for integrated interpretation without structuring the quantitative analyses.

## Results

Twenty Aboriginal adults were recruited. The quantitative analysis included 19 Aboriginal patients (mean [SD] age, 38.4 [15.9] years; 10 women and 9 men) with 1108 documented clinical interactions across emergency, inpatient, and community mental health settings (eTable 1, eMethods in Supplement 1). Seven Aboriginal adults (mean [SD] age, 44.0 [17.8] years; 4 women and 3 men) participated in yarning interviews, with 6 individuals contributing to both quantitative and qualitative components (eTable 1 in Supplement 1).

**Figure 1.**
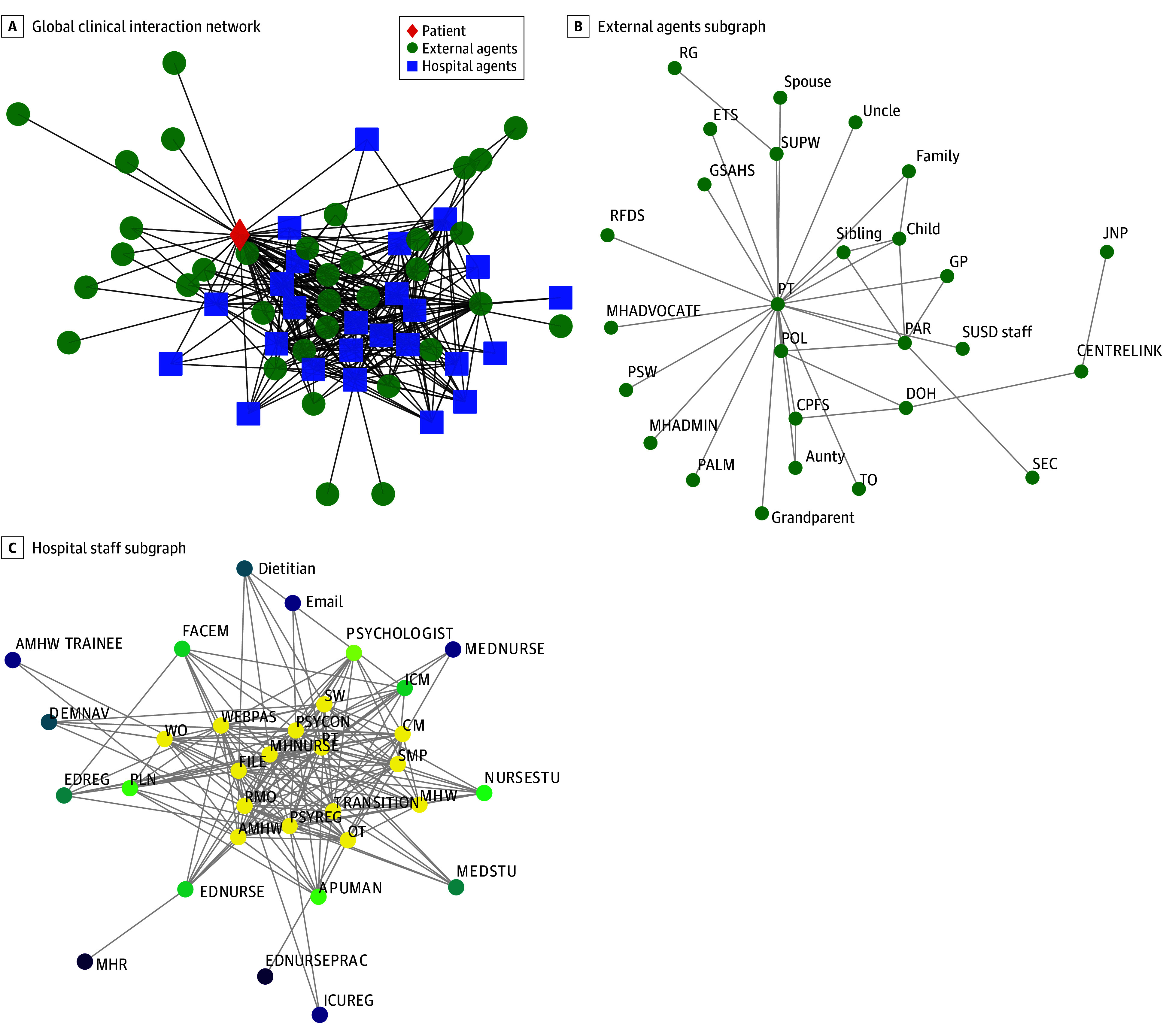
Clinical Interaction Network (CIN) Structure and Induced Subgraphs A, Nodes represent individual agents or information systems; edges indicate co-occurrence within a single recorded clinical interaction. The network demonstrates a core-periphery structure with the patient node centrally bridging 2 largely disconnected groups (external agents and hospital-based personnel), underscoring the dichotomous organization of service delivery pathways within the mental health service. B, Induced subgraph of external agents from the CIN centered on Aboriginal patients (indicated by patient). Edges are unweighted and indicate co-participation in documented clinical interactions. The star-like structure reflects the pivotal role of the patient in connecting disparate external entities, as most external agents link directly to the patient rather than to each other. C, Induced subgraph among hospital clinicians, allied health professionals, AMHWs, trainees, and hospital information systems. Nodes are colored by core-periphery grouping (k-core): yellow nodes indicate the highest coreness (maximal k-core); green and teal, intermediate coreness; and purple, the lowest coreness. Core agents (eg, MHNURSE, PSYCON, PSYREG, AMHW) and core hospital systems (FILE, WEBPAS, MHR) form a radial core-periphery pattern. Edges indicate observed direct/event-based clinical interaction or co-access of information systems during patient care. AMHW indicates Aboriginal mental health worker; APUMAN, acute psychiatric unit team manager; CM, case manager; CPFS, Child Protection and Family Services; DEMNAV, dementia navigator; DOH, Department of Housing; EDNURSE, emergency medicine nurse; EDNURSEPRAC, emergency medicine nurse practitioner; EDREG, emergency medicine registrar; ETS, emergency telehealth service practitioner; FACEM, emergency medicine physician; GSAHS, Great Southern Aboriginal Health Service; GP, general practitioner; ICM, i.Clinical Manager electronic patient information system; ICUREG, ICU registrar; JNP, job network provider; MEDNURSE, medical ward nurse; MHADMIN, mental health administrator; MHADVOCATE, mental health advocate; MHNURSE, mental health nurse; MEDSTU, medical student; MHR, My Health Record; MHW, other mental health worker; NURSESTU, nursing student; OT, occupational therapist; PALM, Palmerston; PAR, parent; PLN, psychiatry liaison nurse; POL, Police; PSW, peer support worker; PSYCON, consultant psychiatrist; PSYREG, psychiatric registrar; RFDS, Royal Flying Doctor Service; RG, rural generalist; RMO, resident medical officer; SEC, security; SMP, psychiatric senior medical practitioner; SUPW, support worker; SUSD, Step Up Step Down; SW, social worker; TO, transport officers; TRANSITION, transition or discharge planning MH nurse; WEBPAS, electronic patient record system; WO, welfare officer.

We constructed a weighted CIN in which nodes represented patients, hospital staff, external agents, and information systems, and connections between nodes were weighted by interaction frequency to reflect how often each pair of people or systems appeared together in care (Figure 1; eMethods in Supplement 1). The aggregated CIN comprised 60 nodes and 327 unique edges, derived from 5845 recorded interactions (Figure 1A; eMethods in Supplement 1). In contrast to prior CINs that showed clear separation between medical and psychiatric teams,^17^ this CIN showed low modularity (M equaling approximately 0.2) and was organized around a central core rather than distinct communities, indicating a small number of structurally central positions within the network (Figure 1; eMethods in Supplement 1). For external agents, the patient node served as the main point of connection with most external roles linked to the patient rather than to each other, producing a star-like structure (Figure 1B). Among hospital staff, connections were concentrated in a densely connected core, with less connected roles positioned around the periphery (Figure 1C).

**Figure 2.**
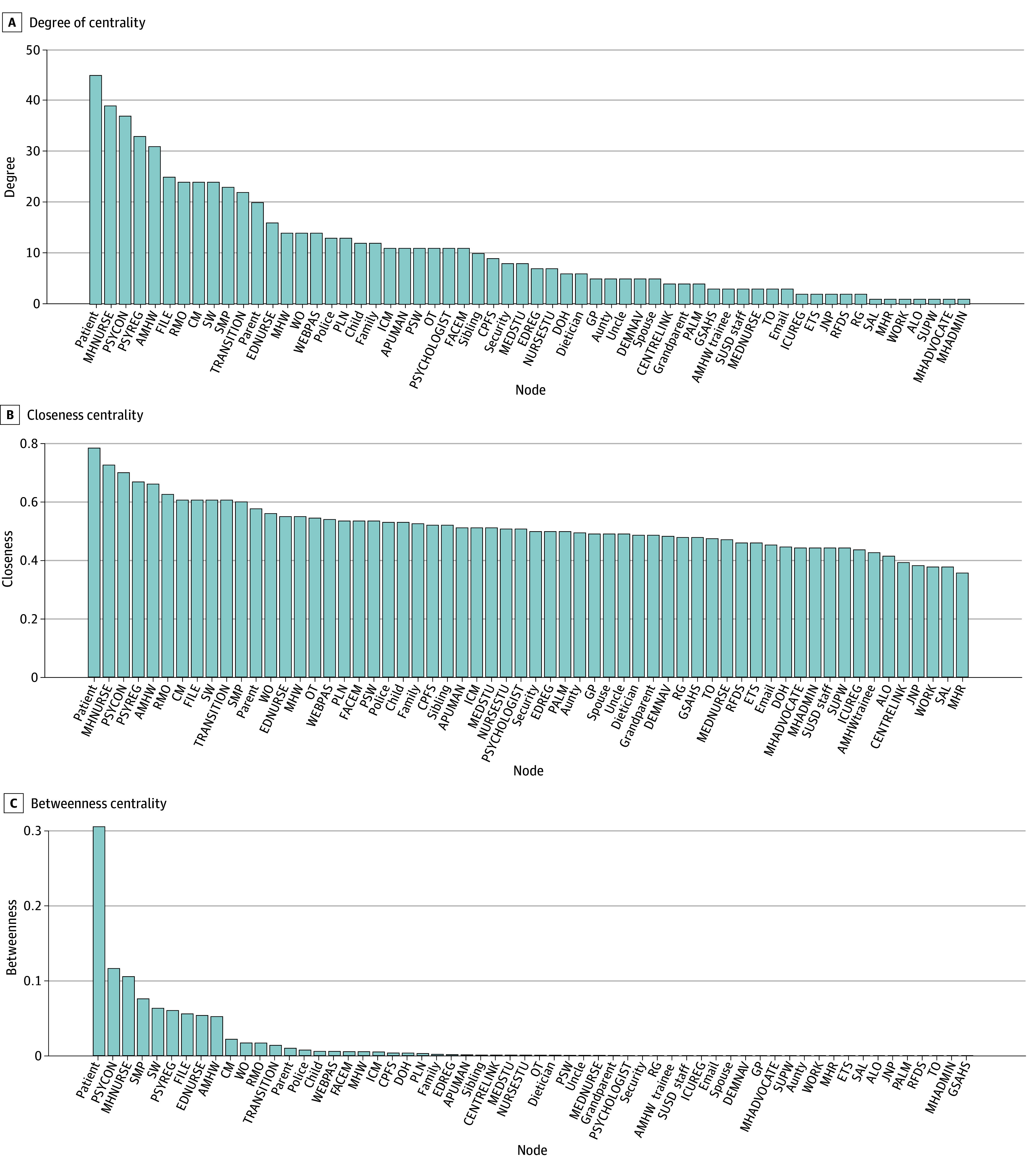
Centrality Measures in the Unweighted Clinical Interaction Network (CIN) for Psychiatric Care Comparative centrality measures for all agents in the constructed CIN, capturing the structure of clinical and organizational relationships among staff, patients, family, and external providers for Aboriginal patients accessing the Great Southern Mental Health Service (GSMHS). The panels display 3 key unweighted centrality metrics calculated across the CIN: A, degree centrality; B, closeness centrality; and C, betweenness centrality. Each agent (node) is represented on the x-axis, ordered and labeled by code, while the y-axis quantifies the corresponding centrality value. The consistently high centrality of MHNURSE, PSYCON, PSYREG and AMHW demonstrates that these positions are organizationally pivotal for information flow, service coordination, and connecting external agents and hospital staff in Aboriginal mental health crisis care. The prominence of FILE in the results reflects its role as the primary data hub. Other hospital staff, external care providers, and trainee roles show lower centrality, indicating more peripheral or specialized positions.

Using unweighted centrality measures, the patient, mental health nurse, psychiatric registrar, psychiatric consultant, and Aboriginal mental health worker (AMHW) were the most prominent roles in the network across degree, closeness, and betweenness, indicating that they were highly connected, centrally located, or positioned between otherwise separate parts of care (Figure 2; eMethods in Supplement 1). The same set of roles remained most prominent in weighted analyses, indicating that these findings were not limited to the unweighted network representation (eFigure in Supplement 1).

We then examined whether the apparent importance of these roles could be explained simply by the number of their connections. Standard double-edge swap randomization was not suitable for this network because it disrupted the organizational structure of the service, so we used the CP-DES approach for surrogate testing (eMethods in Supplement 1).

In these analyses, the consultant psychiatrist and registrar had closeness values at the 18th and 32nd percentiles of the surrogate distribution, and the mental health nurse at the 67th percentile, indicating that their centrality was consistent with what would be expected from their degree and broad structural position alone (eMethods in Supplement 1). By contrast, the AMHW’s closeness centrality exceeded the 99th percentile of the surrogate distribution, demonstrating that this role was not simply well connected, but occupied a structurally distinctive position within the network (eMethods in Supplement 1).

To examine individual care pathways, each patient’s recorded interactions were first encoded as a sequence of 13 standardized action types (such as assessment, intervention, referral, and disposition) arranged chronologically (eMethods in Supplement 1). We then summarized each patient’s pathway as a transition matrix showing how often one action was followed by another (eMethods in Supplement 1). Across all 19 patients, only 5 possible action transitions were not observed, suggesting broad coverage of pathway transitions within the recorded data.

**Figure 3.**
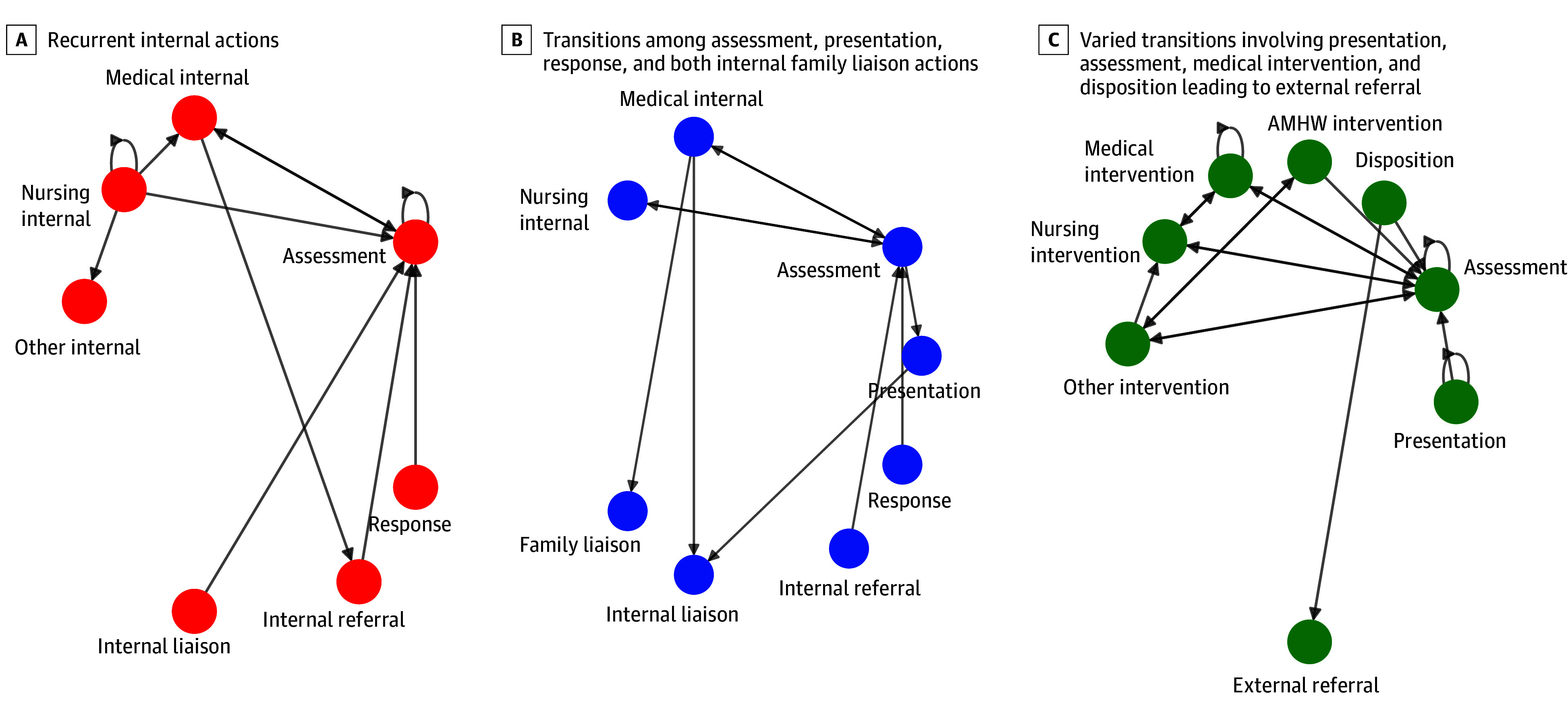
Directed Transition Networks Illustrating Clinical Action Sequence Clustering Directed network diagrams represent characteristic clinical action pathways for 3 clusters of Aboriginal patient trajectories as identified via symbolic sequence analysis and transition matrix clustering. Each node represents a discrete clinical action or event, and directed edges indicate empirically observed transitions between actions within patient care episodes. Position of each node and edge direction reflect action transition structure. See eMethods in Supplement 1 for detailed computational methods and analyses. AMHW indicates Aboriginal mental health worker.

To identify common pathway patterns, we calculated pairwise Jaccard distances between transition matrices and grouped patients using ε-nearest-neighbor network construction (ε = 0.8) with greedy modularity maximization, yielding 6 clusters (Figure 3; eMethods in Supplement 1). Four patients with short or atypical trajectories formed isolated clusters; the remaining 15 grouped into 3 larger clusters, distinguished primarily by how episodes ended (Figure 3). The first pathway was characterized by predominantly internal clinical actions, with episodes concluding in internal follow-up and no progression to external referral. The second reflected prolonged engagement within the service without progression to external referral. The third showed more diverse transitions including pathways from disposition to external referral (often via “other intervention”), suggesting more complex referral processes and possible re-entry into the service system (Figure 3).

### Lived Experiences: Understanding and Acknowledging Aboriginal Ways of Being

**Figure 4.**
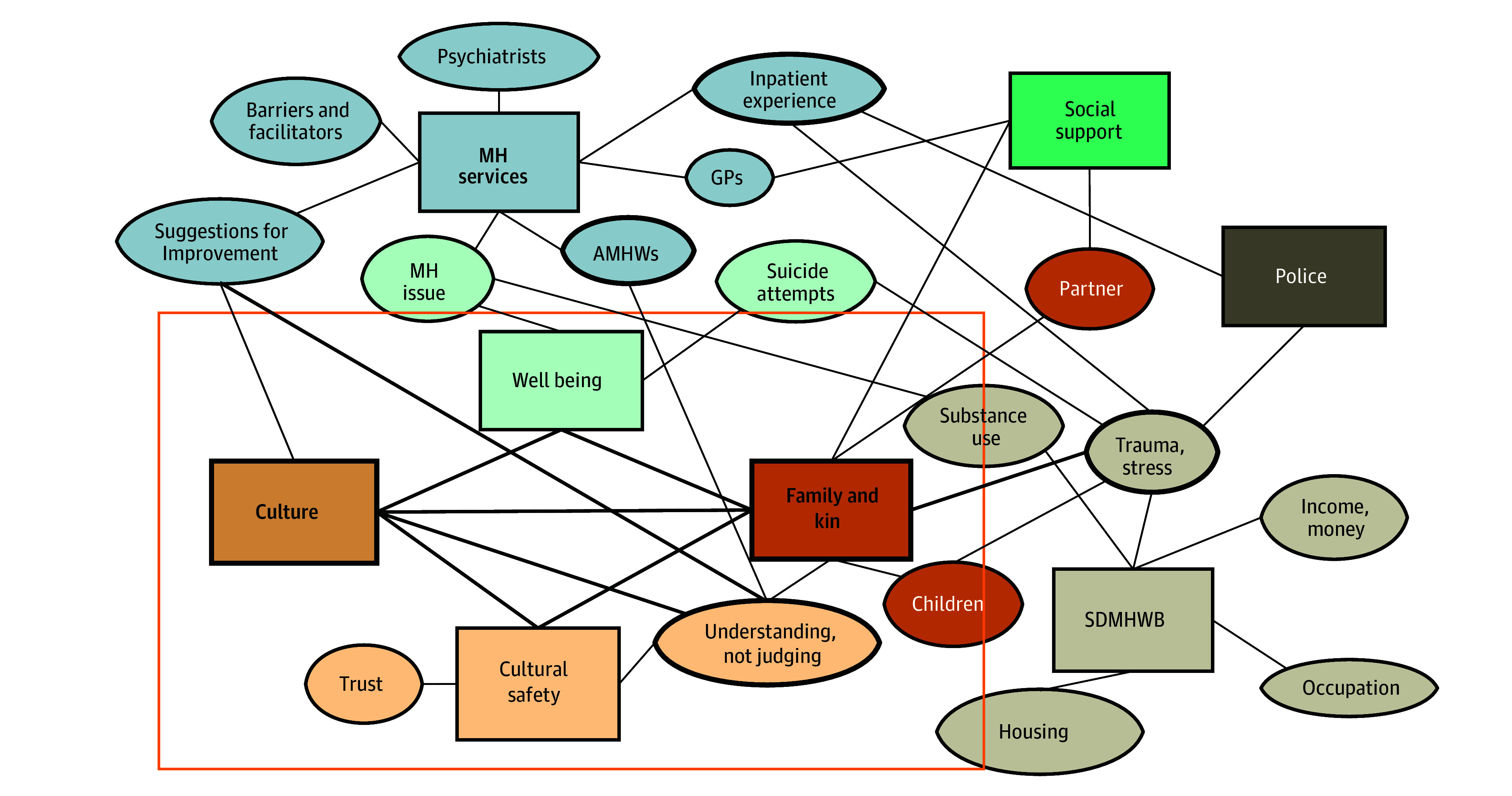
Thematic Network Analysis of Aboriginal Experiences With Mental Health Services Circular nodes represent codes derived from interview data, while square nodes represent overarching categories. Line thickness indicates the strength of connections between themes. The orange box highlights the central interconnected elements of well-being, culture, family and kin, and cultural safety, which form the core of Aboriginal perspectives on mental health care. AMHWs indicates Aboriginal mental health workers; GPs, general practitioners; MH, mental health; SDMHWB, social determinants of mental health and well-being.

Thematic analysis identified 1 overarching theme—understanding and acknowledging Aboriginal ways of being—with 3 interconnected subthemes: (1) the central role of culture in mental health and well-being; (2) the significance of kinship and community; and (3) the impact of trauma and stress (Figure 4). Full participant quotes are available in eTable 5 in Supplement 1.

#### Central Role of Culture in Mental Health and Well-Being

Participants emphasized the profound link between cultural identity, spirituality, and mental health. Connection to country and cultural activities were essential to well-being with many attributing positive experiences at community mental health services to culturally sensitive staff. AMHWs were regarded as vital, although increased staffing and improved cultural training for all staff were requested. In contrast, inpatient environments lacked culturally safe practices, referring specifically to mixed-gender wards. “No, not mixed, because we go to sleep in the same corridor as well as men’s,” said one participant. “That’s against our culture.” Night-time checks with torches were also singled out as culturally unsafe practices: “They point that torch straight in your eyes.…Not only does it wake you up, but the belief and the cultural stuff that we know, that happens.”

#### Significance of Kinship and Community

Kinship and community relationships were central to how participants understood their mental health and their interactions with services. Participants described Aboriginal family systems as extensive, relational, and deeply embedded in obligations, history, and shared experience, yet often poorly understood by non-Aboriginal clinicians. One participant explained that she wanted to speak with an AMHW because “they get it,” saying, “There’s a whole thing there about, yeah, our Indigenous families that…some people just don’t get. Like the size of them and how many funerals we have to go to.” Participants’ accounts showed that kinship could be both a source of strength and a source of distress. Family-related stressors, including bereavement, relationship breakdown, child removal, and long-standing interpersonal conflict, were frequently described as precipitating or worsening mental health crises (eTable 5 in Supplement 1). One participant linked her distress directly to child protection involvement, stating, “DCP [Department for Child Protection] have taken my baby and it’s a 12-month order. So now I don’t have any of my kids with me and I’m just an emotional wreck.”

At the same time, family and kin were also described as vital sources of emotional and practical support. Participants spoke about family as a reason to seek help, a source of encouragement for recovery, and a network that could make engagement with care possible. “I knew something had to change in my life,” said one participant. “Knowing that my children were going to be looked after by my aunty…made all the difference to me.”

Advice from services that overlooked Aboriginal kinship obligations could be experienced as culturally discordant, including suggestions to “cut off” family members. Said a participant, “Cutting them off is not something I want to do. Boundaries, good, moving to a different town, also good.”

#### Impact of Trauma and Stress

Trauma, grief, and chronic stress—stemming from domestic violence, substance use, homelessness, violent deaths and colonization—were pervasive. Suicidal ideation and attempts were common. For example, one participant described, “Not suicidal thoughts but just thoughts of had enough and just wanted to, didn’t want to harm myself but just couldn’t kind of cope there.” Involuntary admissions involving police were especially traumatic, underscoring the protective role of AMHWs: “If [an] AMHW was there straightaway, then I would feel safer…policemen frighten me.…AMHWs would be able to calm us a bit more, have more of an understanding of what’s been going on with us.”

Participant recommendations call for systemic improvements including more AMHW involvement, ongoing cultural and trauma-informed training, support for non-Aboriginal cultural champions, gender-separated wards, and culturally relevant inpatient activities and opportunities for connection to country (eTable 6 in Supplement 1). They envisioned services where Aboriginal cultural experiences are inherently understood. As a participant explained, “Because the spaces that we want don’t exist yet. This place where you can come and just go, ‘I don’t have to explain my whole culture to you. I don’t have to explain anything.’”

### Integration of Qualitative and Quantitative Findings

Joint display-supported integration generated meta-inferences by bringing together Aboriginal peoples’ lived experiences and the quantitative network structure of GSMHS (Table). These integrated findings highlighted the pivotal but inconsistently available role of AMHWs in culturally safe mental health care. Qualitative findings emphasized relational trust, cultural brokerage, and the urgent need for trauma-informed, culturally safe practices and Aboriginal leadership. Quantitative findings demonstrated AMHWs’ central structural position in clinical networks, while identifying risks of role overload and systemic reliance on patients as intermediaries between external agents and hospital staff. Integrated interpretation further highlighted fragmented care, limited incorporation of family and kin within documented workflows, variability in cultural safety among frontline staff, and complex, nonlinear patient trajectories. Together, these meta-inferences underscore the need for sustained workforce expansion, Aboriginal governance, and service redesign centered on embedded cultural safety.

**Table.** Joint Display of Integrated Qualitative and Quantitative Findings

Theme	Qualitative findings	Quantitative findings	Meta-inferences
AMHW role	Participants consistently felt safer and culturally understood when AMHWs were involved. AMHWs described as cultural brokers who “get it.” Desire for increased AMHW presence and early involvement, particularly during ED admissions involving police or ambulance.	AMHW has high degree and closeness centrality in CIN. CP-DES surrogate testing shows AMHW’s closeness significantly exceeded expectations based on degree and community position. AMHWs serve as bridges between hospital staff and external agents.	Qualitative findings emphasized relational trust, cultural safety, and cultural brokerage, while quantitative findings showed that AMHWs occupied a structurally central position in the network. Considered together, these findings suggest that AMHWs play an important role in the cultural and organizational functioning of care.
Inconsistent AMHW presence	Participants reported inconsistent availability of AMHWs during initial crises in the ED. Absence contributed to feelings of cultural unsafety and isolation. Calls for consistent AMHW coverage.	AMHW shows high centrality measures suggesting operational integration, but quantitative data does not capture temporal consistency or adequacy. The network structure implies AMHW burden and potential risk cultural responsibility placed on this role.	Although quantitative findings showed that AMHWs occupied a central structural role in care delivery, qualitative accounts indicated that their presence was not consistent at critical moments. Considered together, these findings highlight the importance of adequate staffing and organisational support for AMHW involvement.
Fragmented care and patient burden	Participants described feeling culturally unsafe when navigating transitions within GSMHS. Calls for trauma-informed and culturally responsive entry points reducing patient burden.	Patient node shows highest betweenness centrality, linking external agents (police, family, GPs) with hospital staff. Structural dependency on patients as intermediaries.	Taken together, the qualitative and quantitative findings highlighted fragmented care pathways and culturally unsafe service experiences, while the network analysis identified patients as occupying intermediary positions across services. These findings suggest the value of culturally embedded navigator roles across phases of care, alongside trauma-informed models aimed at improving continuity and cultural safety.
Frontline staff and cultural safety	Participants noted variability in care delivery: some staff respectful of cultural protocols while others disregarded them. Strong recommendation for ongoing cultural safety training across all staff.	Frontline clinical roles (nurses, psychiatric registrars, consultants, AMHWs) are structurally central in the network . Network structure suggests that the effectiveness and quality of care align with these central frontline roles.	Integrated findings highlight the important role of frontline staff in shaping Aboriginal patients’ experiences of care. Quantitative findings identified several frontline roles as structurally central in the clinical network, while qualitative findings emphasized the need for culturally safe interactions. Together, these findings point to the importance of ongoing cultural safety training and service-wide workforce development.
Family and kin	Family and kin provide emotional and practical support but can also be sources of stress (eg, loss, child custody) due to social inequities. Family involvement essential for healing and cultural identity.	Family members were occasionally involved in clinical interactions as external agents, with family liaisons appearing less frequently and with lower centrality than core clinical staff in the network analysis.	Qualitative findings identified family and kin as central to identity, healing, and distress, whereas quantitative findings showed limited incorporation of family within documented clinical workflows. Taken together, these findings suggest that existing service processes may not adequately reflect the importance of kinship and family relationships in Aboriginal mental health care.
Trauma and nonlinear care journeys	Participants reported nonlinear and unpredictable mental health journeys marked by repeated crises. High trauma burden linked to family loss, intergenerational trauma, systemic racism. Calls for trauma-informed care and enhanced AMHW support.	Transition matrix clustering revealed three main trajectory clusters: Internally focused care and clear internal follow-up Extended in-department engagement Frequent external referrals and repeated admissions No clear correlation between presentation modes and patient trajectories	Qualitative accounts of trauma, crisis, and nonlinear care experiences provided context for the quantitatively identified heterogeneity of patient trajectories. Taken together, these findings suggest that rigid linear models of care may be poorly suited to Aboriginal mental health journeys and point to the importance of trauma-informed, culturally safe, person-centered service design.
Aboriginal roles and leadership	Recommendations include more AMHWs, ongoing cultural-safety training, gender-segregated wards, access to country. Call for authentic Aboriginal governance and transformative change, not tokenistic approaches.	The core-periphery network structure, with AMHWs positioned in the dense inner core alongside other key clinical roles, demonstrates their structural integration into the care system’s information flows	Taken together, the qualitative and quantitative findings highlight the importance of embedded Aboriginal roles and Aboriginal leadership within mental health services. Integrated interpretation suggests that these roles are important to culturally safe care and to how the service system functions.

Abbreviations: AMHW, Aboriginal mental health worker; ED, emergency department; GPs, general practitioners; GSMHS, Great Southern Mental Health Service.

## Discussion

To our knowledge, this study is the first to map Aboriginal mental health care pathways by integrating clinical network analysis and patient trajectory modeling with culturally grounded qualitative analysis within an APAR framework. Together, these methods provided complementary insights into the relational, cultural, and organizational dimensions of psychiatric care that would not have been captured as fully by either approach alone.

A novel finding was that patients occupied central bridging positions within the CIN, linking external agents and hospital staff. This adds to prior network analyses of psychiatric care settings^17^ by identifying the patient not only as the focus of care, but also as a key structural intermediary through whom information and coordination were routed. This is particularly concerning in culturally unsafe or stigmatizing environments and is consistent with evidence of fragmented, discontinuous care for Aboriginal and Torres Strait Islander peoples in acute mental health settings.^8^ Although the network analysis identified patients as key intermediaries within the care system, this did not emerge as an explicit standalone theme in the qualitative interviews, suggesting that system-level coordination demands may become normalized within care experiences and remain less visible without quantitative structural analysis.

Thematic analysis from yarning interviews emphasized the centrality of cultural identity, kinship, and collective trauma in shaping care experiences. Participants expressed strong preference for care involving Aboriginal staff and emphasized cultural breaches in service environments. These experiences align with evidence that culturally unsafe environments contribute to avoidance, trauma amplification, and poor engagement.^19,20^

A key quantitative finding was that the AMHW occupied a statistically exceptional position in the network, with closeness centrality above the 99th percentile in community-preserving surrogate networks. To our knowledge, this is the first study to use surrogate testing to show that the AMHW’s high centrality reflected unique structural connectivity beyond what would be expected from the number of their connections or their general place in the service structure. This finding provides empirical support for qualitative and policy arguments that AMHWs are pivotal to culturally safe care^21^ and adds a new level of structural evidence that strengthens the case for investing in this workforce role.

Qualitative findings highlighted inconsistent AMHW availability during key moments, reflecting an implementation gap. This “cascade vulnerability”^22^ suggests that the absence of culturally safe support may compound existing disadvantages during mental health crises and increase burden on patients and other staff. Together, these findings challenge the view of cultural safety as solely an individual competency and instead demonstrate that it is a structural property of the care system requiring sustained investment, adequate staffing, and organizational embedding.

To our knowledge, this is the first study to characterize distinct care pathway patterns for Aboriginal patients in a regional mental health service. The 3 identified trajectories—internal care with minimal external referral, prolonged internal engagement, and complex external referral with repeated readmission—highlight heterogeneous care and point to transition points where targeted investment could improve coordination and reduce readmissions.

Together, the network, trajectory, and qualitative findings offer a transferable framework for system redesign by: (1) identifying high-leverage roles; (2) identifying structurally central and potentially burdened positions; (3) clustering patient trajectories to match workforce roles with critical transition points; and (4) aligning staffing and investment with observed patterns of influence. These tools can help health systems identify where interaction patterns diverge from formal structures and redesign care pathways accordingly. Policy and practice should prioritize strengthening and sustainably resourcing the AMHW role and mandate cultural safety as a core organizational standard across governance, workforce, and care pathways.

### Limitations

Limitations include sample size of 19 patients for the quantitative component; however, the granularity of 1108 recorded clinical interactions enabled network and trajectory modeling that would not have been achievable with summary-level clinical data. Retrospective collection of interaction data from medical records may have missed informal or undocumented interactions, potentially underrepresenting relational aspects of care pathways. Only 6 participants contributed to both the quantitative and qualitative components, which limited direct participant-level integration. The absence of patient intermediary burden as an explicit qualitative theme may reflect differences in what the 2 components were designed to capture, and future studies should explicitly probe coordination experiences in qualitative interviews to enable direct comparison. Future research should extend this framework to diverse settings, incorporate longitudinal follow-up to examine how care pathways evolve over time, and integrate community-defined outcome measures through participatory evaluation.

## Conclusion

In this mixed-methods study of Aboriginal adults in an Australian mental health system, AMHWs occupied structurally distinctive central bridging positions in surrogate networks while patients served as intermediaries between disconnected system components. Qualitative findings highlighted inconsistent AMHW availability during crises alongside themes of cultural safety, kinship, and trauma. Strengthening culturally grounded roles and redesigning care pathways based on operational information flow may improve care coordination and cultural safety in multisetting mental health systems.

## Notes

Supplement 1. eMethods. eAppendix. Qualitative data analysis eTable 1. Demographic and clinical characteristics of study participants in quantitative and qualitative analyses eTable 2. Reporting of the mixed-methods study in relation to COREQ and GRAMMS criteria eTable 3. Quality appraisal tool for Aboriginal and Torres Strait Islander studies: evaluation of our study's criteria fulfillment eTable 4. Yarning guide for the qualitative component of the study eTable 5. Participant quotes illustrating key themes from qualitative interviews eTable 6. Characteristics of culturally safe mental health services and proposed strategies for implementation in a community mental health service and inpatient unit eFigure. Weighed centrality measures of the Clinical Interaction Network eReferences.

Supplement 2. Data Sharing Statement
