# Supplementary material for: Mapping Aboriginal Mental Health Journeys Through Psychiatric Care Systems
Source: JAMA Netw Open. 2026 May 20;9(5):e2613904. doi: 10.1001/jamanetworkopen.2026.13904 (PMC13191385; doi:10.1001/jamanetworkopen.2026.13904)
Supplement: Supplement 1. — eMethods. eAppendix. Qualitative data analysis eTable 1. Demographic and clinical characteristics of study participants in quantitative and qualitative analyses eTable 2. Reporting of the mixed-methods study in relation to COREQ and GRAMMS criteria eTable 3. Quality appraisal tool for Aboriginal and Torres Strait Islander studies: evaluation of our study's criteria fulfillment eTable 4. Yarning guide for the qualitative component of the study eTable 5. Participant quotes illustrating key themes from qualitative interviews eTable 6. Characteristics of culturally safe mental health services and proposed strategies for implementation in a community mental health service and inpatient unit eFigure. Weighed centrality measures of the Clinical Interaction Network eReferences. [file jamanetwopen-e2613904-s001.pdf]

Supplemental Online Content

Milroy H, Banushi B, Briand BC, et al. Mapping Aboriginal mental health journeys through psychiatric care systems. *JAMA Netw Open.* 2026;9(5):e2613904. doi:10.1001/jamanetworkopen.2026.13904

eMethods.

eAppendix. Qualitative data analysis

eTable 1. Demographic and clinical characteristics of study participants in quantitative and qualitative analyses

eTable 2. Reporting of the mixed-methods study in relation to COREQ and GRAMMS criteria

eTable 3. Quality appraisal tool for Aboriginal and Torres Strait Islander studies: evaluation of our study's criteria fulfillment

eTable 4. Yarning guide for the qualitative component of the study

eTable 5. Participant quotes illustrating key themes from qualitative interviews

eTable 6. Characteristics of culturally safe mental health services and proposed strategies for implementation in a community mental health service and inpatient unit

eFigure 1. Weighed centrality measures of the Clinical Interaction Network

eReferences.

This supplemental material has been provided by the authors to give readers additional information about their work.

© 2026 Milroy H et al. *JAMA Network Open*.

eMethods

Construction of the Clinical Interaction Network

To construct the Clinical Interaction Network (CIN) we initially followed the methodology outlined by McCullough et al1

An undirected graph, G, is created from a vertex set, V, where each $i \in V$ represents an agent or information system. Edges between two vertices i and j are constructed if the agents or information systems are present in the same interaction instance. However, edges must observe additional criteria to exclude certain forbidden edges. Information systems cannot be accessed directly by other information systems and can only be accessed by relevant clinical staff. For example, the My Health Record (MHR) is only accessible by agents belonging to the psychiatric team. Thus, edges involving information systems are restricted only to (a) agents that (b) have the appropriate clearance. eMethods Table 1 and eMethods Figure 1a illustrate this construction scheme with an example event log.

eMethods Table 1. Example event log used in the construction of the Clinical Interaction Network (CIN). This table provides an excerpt of the de-identified observational dataset collected at the Great Southern Mental Health Service (GSMHS), illustrating the key variables used to build the CIN. Each row represents a single recorded clinical interaction, identified by Patient ID and timestamped with date and time of occurrence. The Agents column lists the individuals or roles (e.g., PT = Patient; MHNURSE = Mental Health Nurse; CM = Case Manager) involved in the interaction, while the Information Systems column specifies any electronic or physical health record systems accessed during that encounter (e.g., FILE = patient file). Interactions may include direct patient contact, liaison activities, assessments, or interventions, and can involve multiple agents and systems in the same event. This format was applied consistently across the full dataset (N = 1108 interaction instances) to define network nodes and edges for subsequent CIN analysis, with edges representing co-occurrence of agents/systems within an interaction

Patient ID	Date	Time	Agents	Information Systems
1	31-01-2024	1230	MHNURSE, PT	FILE
1	31-01-2024	1430	CM, PT	
1	02-02-2024	0830	MHNURSE, CM	FILE
1	02-02-2024	0930	MHNURSE, PT	

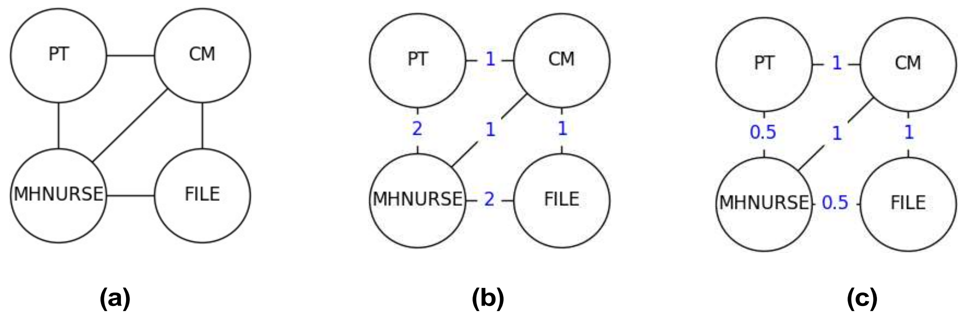

eMethods Figure 1: Example construction of a Clinical Interaction Network (CIN) from a sample event log (eMethods Table 1). (a) Unweighted CIN: nodes represent agents or information systems with edges indicating co-occurrence in the same interaction instance. (b) Frequency-weighted CIN: edge weights represent the total number of recorded interactions between each pair of nodes. (c) Distance-weighted CIN for path-based analysis: interaction counts are converted to edge distances using the reciprocal of frequency, so that more frequently interacting nodes are separated by shorter distances for shortest path and path length calculations. Abbreviations: PT = Patient; MHNURSE = Mental Health Nurse; FILE = Patient file in the health record system; CM = Case Manager

While this construction mirrors the approach by McCullough et al. [1], our methodology includes additional information into the network model due to a more extensive dataset. McCullough et. al.[1] analysed a total of N = 213 interactions, whereas our dataset contains N = 1108 interactions, allowing edges to be defined with weights corresponding to the frequency of interactions. Consequently, a more nuanced analysis is available, distinguishing

© 2026 Milroy H et al. *JAMA Network Open*.

between frequent and infrequent interactions. The unweighted CIN represents all possible interactions, while the weighted CIN represents how frequently these interactions occur.
To use weighted centrality measures that utilise weighted path lengths and shortest distances, a notion of distance between nodes is required. Interactions with high frequencies should correspond to shorter path lengths, and interactions with low frequency should correspond to longer path lengths. Thus, we define the distance between two nodes as the reciprocal of interaction count. eMethods Figures 1b and 1c demonstrate the extension of McCullough et al[1].'s CIN model with two additional layers of interaction frequency and distance.
This construction scheme minimises external assumptions, only limiting interactions to maintain feasible interactions from an organisational standpoint. Nonetheless, some limitations exist due to the data collection process
To collect data with minimal impact on clinical outcomes, interactions were observed with no additional clinical information that may inform the severity or significance of an interaction. While the action field may be used to give weighting to each interaction, we assume that each observed interaction is of equal weighting for simplicity. Thus, our model is only able to describe structural features based on what interactions are present and the frequency they occur, not the nature of the interaction.
Events with a large number of agents, such as team meetings, are unlikely to have every agent interacting between each other equal significance. Information about individual interactions is lost for simplicity in data collection. The network model assumes that every possible interaction occurs with equal significance, thus over-representing the total number of interactions present. Similarly, information systems may exhibit an artificially higher connectivity as we assume that every agent capable will always access an information system. eMethods Table 2 and eMethods Figure 2b illustrate this effect with an example interaction instance. eMethods Figure 3 shows that most interaction instances have two or fewer agents per interaction instance. Thus, the over-representation of interactions is minimal and unlikely to impact the overall structure of the CIN significantly.

eMethods Table 2. Example event log entry illustrating potential over-representation of interactions in clinical communication networks. This example shows a single logged instance in which MHNURSE conducts a routine patient observation and simultaneously accesses the patient's file. Concurrently, an AMHW and a RMO are present to inform the patient of a discharge plan. Although these are separate tasks with distinct purposes, standard event logging records them within the same time frame and may therefore capture them as a single interaction. In network analyses that do not distinguish between task context or communication intent, such an event may artificially inflate the perceived strength of connections between staff roles. This highlights the importance of filtering and categorizing event logs to avoid misinterpretation of collaboration patterns.
Abbreviations: PT = Patient; MHNURSE = Mental Health Nurse; RMO = Resident Medical Officer; AMHW = Aboriginal Mental Health Worker; FILE = Patient file in the health record system

Patient ID	Date	Time	Agents	Information Systems
1	31-01-2024	1230	PT, MHNURSE, RMO, AMHW	FILE

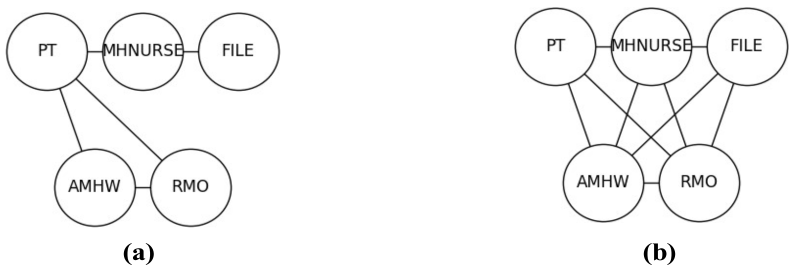

eMethods Figure 2. Comparison of theoretically expected vs. modeled CINs from an example event log interaction. (a) CINs that are expected to arise from the interaction described in e eMethods Table 2, based on the assumption that all temporally co-occurring events represent a potential link between participating agents. In this example, the agents are PT, MHNURSE, RMO, and AMHW, with FILE representing the patient's electronic medical record. All possible dyads implied by the raw interaction data are shown, assuming equal significance of all co-present agents. (b) CINs generated using our construction scheme, which applies feasibility constraints such as restricting information system access to relevant clinical staff and assuming complete interaction between all eligible agents within an event. These methodological decisions can remove or add edges compared to the idealized model in (a). Differences

© 2026 Milroy H et al. *JAMA Network Open*.

between (a) and (b) illustrate how construction rules affect inferred connectivity and consequently network metrics. Abbreviations: PT = Patient; MHNURSE = Mental Health Nurse; RMO = Resident Medical Officer; AMHW = Aboriginal Mental Health Worker; FILE = Patient file in the health record system.

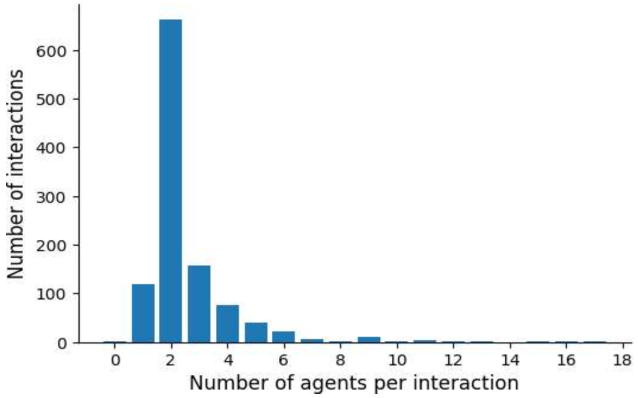

eMethods Figure 3. Distribution of agents per clinical interaction. Histogram of 1,108 recorded interactions for 19 Aboriginal patients at the GSMHS showing the number of agents involved per event. Most interactions involved one or two agents - typically the patient and a single clinician - with progressively fewer multi-agent events. This pattern indicates a predominance of dyadic encounters and minimal inflation of pairwise links from multi-party events in constructing the CIN. Agent categories include patients, clinical staff, external providers, and family members. x-axis = number of agents; y-axis = count of interactions.

Surrogate Testing

We introduce the main ideas motivating surrogate testing to detect nonlinearity in time series analysis [2,3]. A surrogate ensemble is produced that preserves certain characteristics of the original time series (such as the mean or variance). These surrogates are constructed using either typical or constrained realizations. Typical realizations preserve characteristics by estimating parameters of the original time series and use a generative process to form surrogate data reflecting these parameters. This is in contrast to constrained realizations which contain exactly preserved characteristics by modifying the original process, typically from randomization and shuffling algorithms.

An appropriate discriminating statistic sensitive to non-linearity is then applied to the surrogate ensemble and original time series. In the framework of hypothesis testing, the null hypothesis being scrutinized asks whether the nonlinearity of the time series can be attributed solely to randomization. If the original time series is indeed statistically significant, the null hypothesis is rejected, meaning that the randomization with preserved characteristics cannot explain the nonlinear behavior. If the original time series is not significant, the null hypothesis cannot be rejected [3].

By starting with the simplest null hypotheses (such as random noise) and working towards increasingly complex surrogates, a hierarchy of null hypotheses can be created that iteratively exclude specific characteristics as being explanatory for the nonlinear behavior of the time series [2]. Here, we extend this idea from time series to graphs and apply it to CINs. That is, we start from an observed graph and create an ensemble of randomized graphs. From that ensemble we estimate certain properties of the nodes of each graph and ask whether the original graph has properties typical of that ensemble.

Graph randomization

The first typical realization scheme available is the Erdős-Rényi model which requires an edge probability parameter p to be estimated from the original network [4]. By specifying the number of nodes, each edge is then constructed with probability p to produce networks with binomial degree distribution. To produce networks with a prescribed degree sequence, the configuration model may be used [5]. This model works by initializing nodes with half-edges corresponding to the degree sequence. Two nodes are then chosen at random to connect their two half-edges until all half-edges are resolved. To maintain uniform sampling of nodes, half-edges may be connected to form self-loops and multi- edges. Self-loops and multi-edges may be removed to produce a simple graph, but the resulting degree sequence is no longer exact.

A constrained alternative to the configuration model is the double-edge swap (DES) which modifies the original network by shuffling edge connectivity [6]. This algorithm first selects two edges with distinct endpoints (i, j) and (u, v). Node pairing is then swapped to generate two new edges (i, u) and (j, v). The two old edges (i, j) and (u,

v) are removed with new edges (i, u) and (j, v) inserted if both edges are not already present in the graph. If either (or both) edges already exist, the swap is not performed and new random edge pair is selected instead. Since node degree is preserved at each step, the DES preserves both degree sequence and node degree. An additional constraint is also typically enforced to ensure connectivity of the network is maintained in each swap. Similarly to McCullough et al. [1], we can also impose additional criteria to maintain the constraints in agent and information system interactions for use on the CIN.
Using this scheme on the unweighted CIN, we can interpret randomized surrogates as alternate CINs representing randomized interactions. Considering the weighted CIN, surrogates can be generated by performing the DES and randomly reassigning edge weights. Surrogates are interpreted as networks with randomized communication channels and frequency of interactions. Ideally, the total interaction frequency of each node, represented by the node strength, should be preserved to maintain realism in CIN interpretation. However, attempts to preserve global node strength were unsuccessful and only local node strengths of specified nodes could be achieved. Therefore, we only looked at surrogate testing on the unweighted CIN.

Surrogate 1: The Double-Edge Swap
We used the surrogate testing to examine whether important agents in the CIN have favorable position due to their specific connectivity. We chose closeness to measure agent importance as it is robust to small perturbations in connectivity, making differences in closeness values indicative of major structural differences [7]. Using 500 randomly generated networks, each with 2000 successful edge swaps, closeness values of the Mental Health Nurse, Consultant Psychiatrist, Psychiatric Registrar and AMHW are tested against the original CIN. Since the degree of each node is identical in the surrogate ensemble, the null hypothesis for this test stipulates that high closeness of the important nodes can be fully attributed to high degree. eMethods Figure 4 shows that all four agent nodes have only slightly lower closeness values than each surrogate on average and were thus nonsignificant at any level – when quantified with this measure (closeness). Therefore, the null hypothesis cannot be rejected.

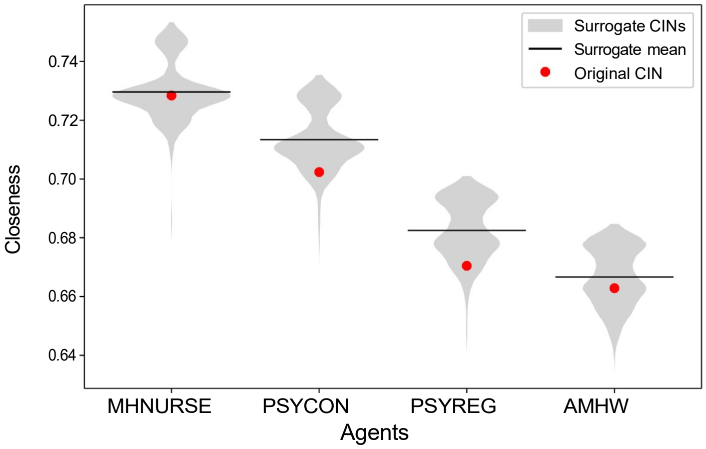

eMethods Figure 4. Violin plots of closeness centrality for key clinical agents. Violin plots show the distribution of closeness centrality from 500 surrogate Clinical Interaction Networks (CINs) generated using the connected Double-Edge Swap (DES) algorithm with 2000 successful swaps. Results are shown for the Mental Health Nurse (MHNURSE), Consultant Psychiatrist (PSYCON), Psychiatric Registrar (PSYREG), and Aboriginal Mental Health Worker (AMHW), who were identified as important agents in the original CIN. Horizontal markers indicate the mean of each surrogate distribution (black line), and the value for the original CIN is overlaid (red point). For all four agents, the original CIN values lie within the surrogate distributions and are consistently lower than the surrogate means, indicating that their observed closeness can be explained by degree alone under this null model. However, because the DES randomization disrupts the intrinsic separation between external agents and hospital staff - producing surrogate networks with more direct connectivity across these groups - the DES constitutes a poor choice of surrogate model for the CIN.

While the surrogate networks have less structure than our original CIN, they are also subject to fewer restrictions on connectivity. Thus, surrogates will not exhibit the same disconnectivity between external agents and hospital staff. Agents in the surrogate ensemble need not disperse information through the Patient node and may connect directly to hospital staff, reducing average path length and explaining the higher closeness. Hence, this surrogate test is flawed as the DES produces networks that are infeasible when considering limitations in how agents may interact. We thus required an alternate network randomization scheme that can preserve these

© 2026 Milroy H et al. *JAMA Network Open*.

structures.

Community Preserving Connected Double-Edge Swap
The DES could be modified with additional constraints to preserve the intrinsic separation between external agents and hospital staff. However, we aimed to develop a modified algorithm that can be applied to other networks more generally. Namely, we describe an algorithm that preserves the number of connections within and between node groups. While the groups found in the CIN were not communities, we call this algorithm the community preserving double-edge swap (CP-DES) to highlight its potential use for networks with strong community structure.

Currently, there is an absence of graph randomization schemes that preserve global community structure. The most similar existing study proposed a method for generating community preserving attribute graphs to reflect online social networks. However, this study aimed to produce accurate depictions based on online social network data rather than a randomization scheme. Another study examined hypergraph randomization schemes that preserved local clustering structure, which is limited to preserving local connectivities rather than global community structures. These distinctions make the CP-DES algorithm, which uniquely focuses on both randomization and community preservation, the first of its kind.

Given a graph G with communities $8\,_{n=1}^{n=N}$ partitioning the vertex set, we define the block network B, which has nodes representing each community C_n with weighted edges between nodes representing the number of edges that span between communities.

The algorithm similarly starts by selecting two edges from G given as (i, j) and (u, v). Each node belongs to a community denoted as Ci, Cj, Cu and Cv. While we picked edge pairs to guarantee each node is distinct, we placed no restriction on the uniqueness of each community. The two selected edges are represented in the block network B as the edges (Ci, Cj) and (Cu, Cv). We replaced the selected edge pair with a new edge pair (i, u) and (j, v) if the edge pair is not already present in the network. Additionally we ensured that connectivity between and within communities before and after the swap remains the same. Namely, the edges (Ci, Cj) and (Cu, Cv) must both be accounted for by the edges (Ci, Cu) and (Cj, Cv). We simply check that after the edge swap the swap has exchange edges within and between the same communities. eMethods Figure 5 illustrates several examples of valid and invalid swaps with this community preserving criterion. Since community connectivity is preserved, graph connectivity is also preserved, so the additional connectivity constraint of the DES is absorbed into the community preserving criterion. If any of the two criteria is invalid, a new edge pair is selected until the prescribed number of edge swaps is reached. Since intra-community and inter-community edges are preserved at each step, the algorithm preserves the connectivity of the block network, thus preserving community structure.

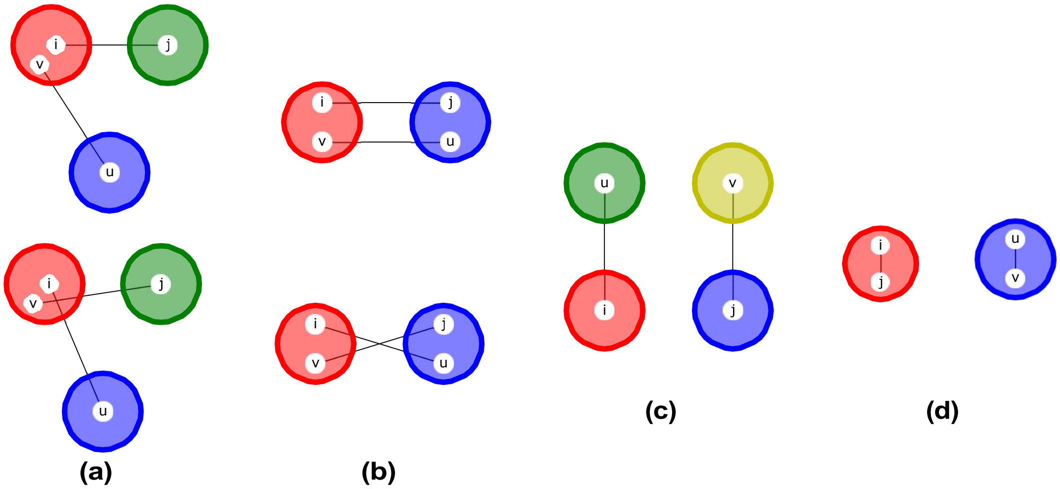

eMethods Figure 5. Illustration of community-preserving double-edge swaps.
(a) Example with three distinct communities (denoted by differently colored node groups) where two edges are rewired in a way that maintains the original distribution of intra-community and inter-community edges. (b) Similar example with two communities, showing a valid swap that preserves the structural relationships between communities. (c) Example with four communities where selected edge pairs cannot be validly swapped without altering the community structure, resulting in an invalid configuration. (d) Example with two communities where a proposed swap is invalid because it changes the proportion of edges connecting nodes within versus between communities. Nodes are colored according to community membership, and solid lines represent edges before swapping, while dashed lines represent rewired edges. A swap is considered “valid” only if the rewiring does not change the number of edges within each community or between any pair of communities.

A synthetic network was generated with community structure to illustrate and to test the algorithm, as shown in eMethods Figure 6a. DES and CP-DES are then used to generate two randomized networks before using maximum modularity to detect communities. eMethods Figure 6b shows that CP-DES randomizes local connectivity while preserving community structure. Contrarily, eMethods Figure 6c shows DES destroys community structure with a low modularity value of $M = 0.25$.

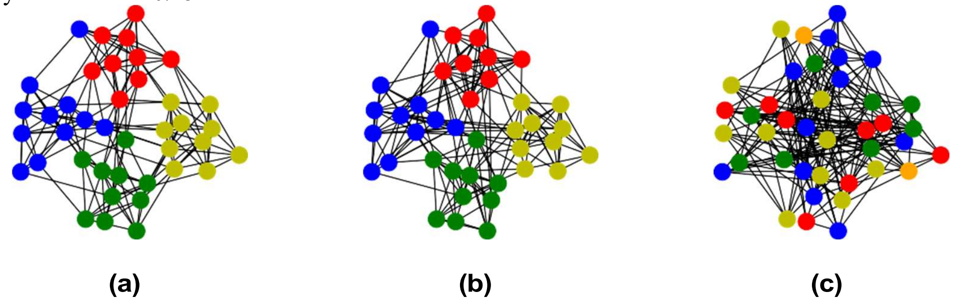

eMethods Figure 6. Synthetic network randomization comparison. (a) Synthetic network with four intrinsic communities (modularity M = 0.48) used to illustrate the effect of different randomization schemes on community structure. Node colors denote community membership; edges represent connections within and between communities. (b) Example of the proposed Community Preserving Double-Edge Swap (CP-DES) algorithm applied to the synthetic network with 1000 successful swaps (M = 0.48). The CP-DES randomizes local connectivity while exactly preserving the number of intra- and inter-community edges, thereby maintaining the original community structure. (c) Standard Double-Edge Swap (DES) applied to the same network with 1000 successful swaps (M = 0.25). The DES preserves the degree sequence but not the between-/within-community connectivity, leading to the destruction of global community structure and markedly reduced modularity.

Surrogate 2: The Community Preserving Double-Edge Swap

We partitioned the CIN into artificial communities. Namely, C1 contains external agents, excluding the Patient; C2 contains the Patient, Mental Health Nurse, and Patient File; C3 contains hospital staff with a core number of 10; and C4 contains the remaining hospital staff with a core number less than 10. Using these communities, the CP-DES generates 500 surrogate networks with 1000 successful edge swaps. These surrogates thus represent randomized CINs that maintained feasible organizational and hierarchical structures but otherwise have randomized connectivity. The original CIN is compared to the surrogate ensemble using the closeness centrality of the most important hospital staff, as shown in eMethods Figure 7. The Consultant Psychiatrist and Registrar have closeness values less than the surrogate networks (18th and 32nd percentile), whereas The Mental Health Nurse has higher closeness (67th percentile). Therefore, as all three agents had non-significant closeness, we cannot reject the null hypothesis that degree and community position can explain their high importance. However, AMHW has closeness significantly higher than in the surrogate networks (top 99th percentile), suggesting that their high closeness cannot be attributed solely to their degree and positions in their respective communities. Therefore, we surmise that the unique connectivity of the AMHW is responsible for their high closeness.

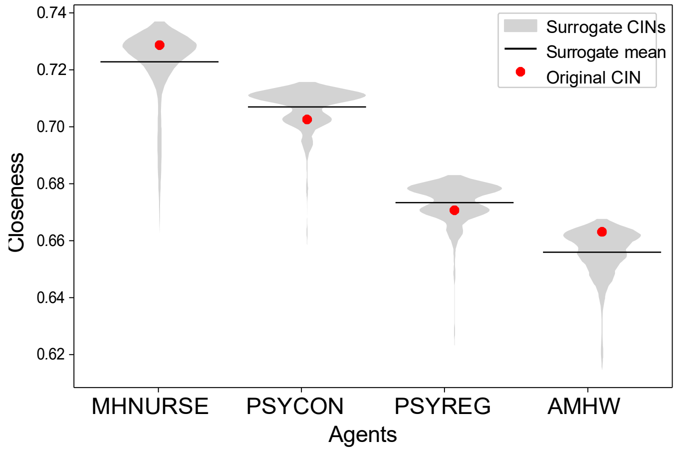

eMethods Figure 7. Distribution of closeness centrality for key clinical roles in the community-preserving double-edge swap (CP-DES) surrogate networks. Violin plots show the distribution of closeness values

© 2026 Milroy H et al. *JAMA Network Open*.

computed from 500 CP-DES surrogate networks (each generated by performing 1000 successful edge swaps while preserving degree sequence and inter-community connectivity). Grey horizontal bars indicate the mean surrogate closeness for each role; black horizontal lines show the observed closeness in the CIN. The Consultant Psychiatrist (PSYCON) and Psychiatric Registrar (PSYREG) had closeness values at the 18th and 32nd surrogate percentiles, respectively, and the Mental Health Nurse (MHNURSE) at the 67th percentile - none significantly different from the surrogate ensemble. In contrast, the Aboriginal Mental Health Worker (AMHW) exceeded the 99th percentile, indicating a level of centrality not explained by degree or community position alone. The AMHW's observed value was a clear outlier relative to its surrogate distribution (cf. eMethods Figure 4).

Symbolic Sequence Construction and Analysis

The most relevant entries to represent the clinical trajectories of each patient are the Interaction and Outcome columns. These columns describe the actions initiating each event and the actions that arose after that. Actions in both columns were course-grained into thirteen distinct symbols, as shown in eMethods Figure 8. We hereon refer to these as action symbols. Events occur irregularly in time as clinical interactions do not occur at regular intervals but rather only when clinical events occur. While trajectories may be modelled as irregularly sampled time series, we disregard the varying time intervals between each symbol and model each trajectory as symbolic sequences. Rather than examining global temporal patterns and timings between actions, we focus on local temporal patterns in action symbol transitions. We hypothesize that the existence and non-existence of particular action symbol transitions allow for the categorization of patient trajectories.

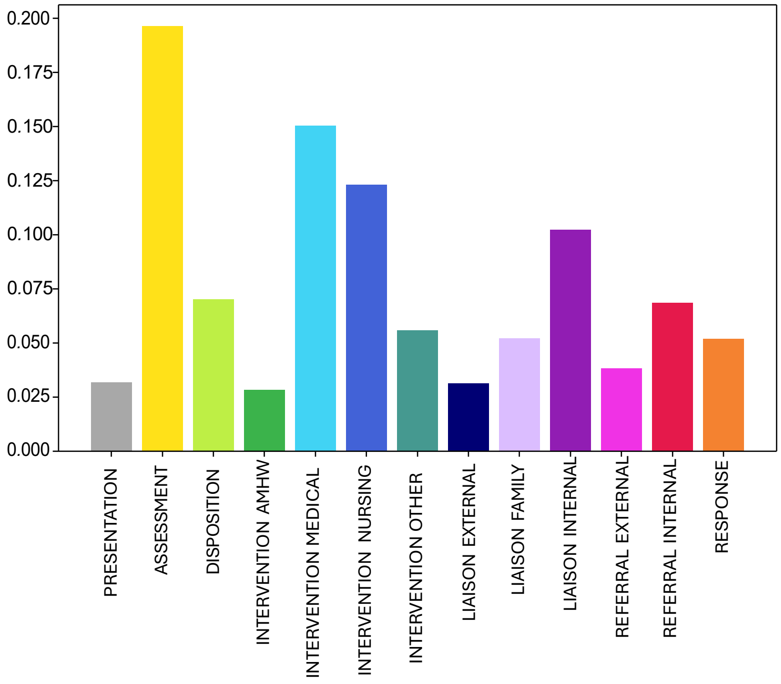

eMethods Figure 8. Probability distribution of action symbols across all recorded clinical interactions. The histogram displays the overall relative frequencies of the 13 coarse-grained action categories derived from the Interaction and Outcome fields in the event log for the 19 observed Aboriginal mental health patients at the GSMHS. Action labels are presented on the x-axis (PRESENTATION, ASSESSMENT, DISPOSITION, INTERVENTION AMHW, INTERVENTION MEDICAL, INTERVENTION NURSING, INTERVENTION OTHER, LIAISON INTERNAL, LIAISON EXTERNAL, LIAISON FAMILY, REFERRAL INTERNAL, REFERRAL EXTERNAL, RESPONSE), with bar heights indicating their relative frequency. This distribution informed subsequent entropy and transition-matrix analyses.

Since data collection was transcribed chronologically, the two action columns were concatenated and flattened to produce a single sequence of action symbols. eMethods Figure 9 demonstrates this process. In doing so, entries with multiple interactions or multiple outcomes may lose causal information linking the two columns. For instance, two inter- actions and two outcomes within a single event should all exhibit transitions for a total of four transitions. However, our model only captures the single transition between the last interaction and the first outcome recorded. eMethods Figure 10 shows that most events contain one interaction and one outcome,

© 2026 Milroy H et al. *JAMA Network Open*.

justifying the small effect of this assumption.

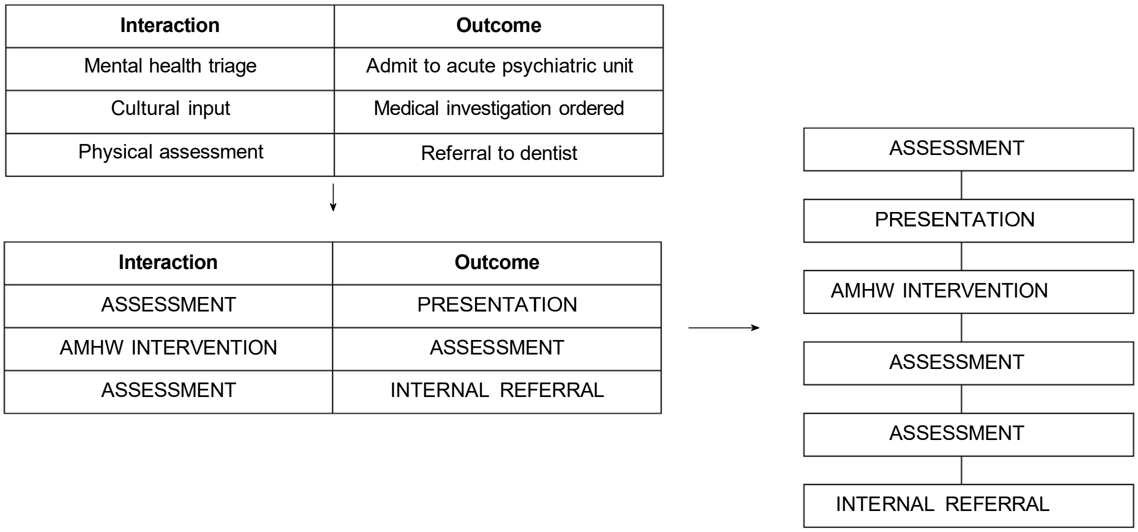

eMethods Figure 9. Symbolic sequence construction scheme. Actions documented in the Interaction and Outcome columns of the clinical event log are course-grained into thirteen distinct action symbols (e.g., Assessment, Presentation, Nursing Intervention, Referral). The resulting symbols from both columns are concatenated chronologically and flattened into a single sequence to represent each patient's clinical trajectory. This transformation captures local temporal patterns in clinical pathways by encoding transitions between successive actions. While causal information linking multiple interactions and outcomes within a single event may be partially lost, this process provides a consistent framework for symbolic sequence analysis and subsequent construction of transition matrices across patients.

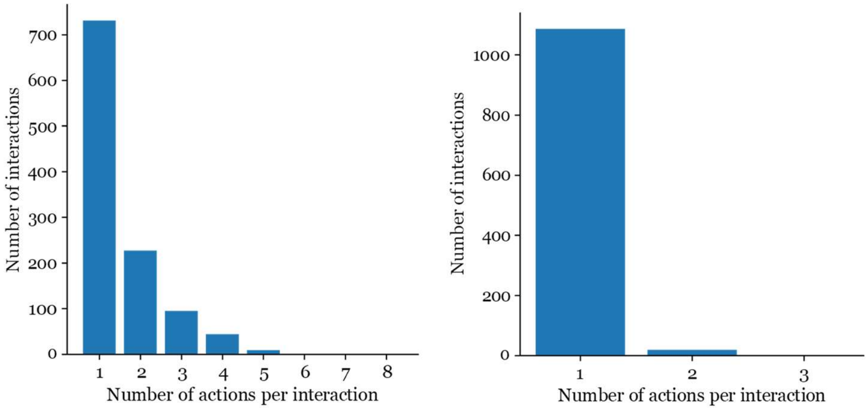

eMethods Figure 10. Histograms of action count in the (a) interaction and (b) outcome columns. Histograms summarizing the number of recorded actions per event log entry for the Interaction and Outcome columns in the GSMHS dataset. Each event instance was coded into one or more coarse-grained action symbols representing presentations, assessments, interventions, liaison, referrals, disposition, and response behaviors. The majority of events contained a single recorded action, reflected in the peak at one action per entry in both distributions. A smaller proportion of entries contained two actions with very few involving three or more. This indicates that most clinical interactions were documented as a simple one-to-one mapping between an initiating action and an outcome, supporting the simplifying assumption used in symbolic sequence construction (i.e., concatenating Interaction and Outcome into a single sequence). These distributions suggest minimal over-representation of transitions was introduced by the modelling approach.

Due to the lack of global temporal information in our symbolic sequence, methods such as symbolic recurrence analysis and symbolic dynamic time warping are unavailable [9,10]. Instead, we looked at probabilistic and statistical methods to analyze our symbolic sequences. Information theory is frequently used to quantify the

© 2026 Milroy H et al. *JAMA Network Open*.

complexity and uncertainty within a time series [11]. By considering the probability distribution of symbols p(x) as a random variable X over the symbolic alphabet $x \in X$, time series are represented by their associated probability distributions. Characteristics of that distribution is quantified by the Shannon entropy, given by:

$$H(X) = -\sum_{x \in X} p(x) \log(p(x)).$$

Intuitively, probability distributions have low entropy when probability distributions are heavily skewed towards a particular value, reducing the uncertainty in observation. Contrarily, more uniformly dispersed distribution have higher entropy as each symbol is equally likely to be observed.

We utilized Ferriera and Zhao's community detection technique for its ability to create arbitrarily shaped clusters [12]. Ferriera and Zhao propose *k*-nearest neighbors and ε-nearest neighbors for network construction. Both methods are shown to work accurately with ε-nearest neighbors performing marginally better. Thus, we used ε-nearest neighbors to take only edges between nodes if their distance is less than the minimum threshold ε. Greedy modularity maximization community detection is used to cluster nodes. Each symbolic sequence is used to construct its probability distribution, with the entropy of each distribution displayed alongside a visualization of the corresponding symbolic sequence in eMethods Figure 11.

Looking at the entropy values shown in eMethods Figure 11, one significant observation is the variation in predictability across patients. For example, Patient 9 has a lower entropy, indicating a more predictable trajectory dominated by a smaller subset of service actions. In contrast, Patients 1 and 18 exhibit higher entropy, reflecting a broader and more diverse range of action symbols. eMethods Figure 11 reflects this observation as Patient 9 has lower entropy, while Patients 1 and 18 have higher entropy. Generally, entropy correlates with symbolic sequence length for sequences less than 100 symbols. This likely suggests that short sequences have low entropy due to their inability to fully populate their probability distributions.

For the six trajectories with significant size, namely Patients 1, 7, 9, 10, 13 and 14, entropy indicates the predictability of each distribution. Distributions with low entropy, such as Patient 9, are more predictable as they require a smaller subset of service actions. Contrarily, distributions with high entropy, such as Patient 14, require a broader, more holistic spectrum of action types. However, this notion of predictability does not necessarily correlate with the actual predictability of a patient's trajectory. For instance, patients with more uniform distributions may still have predictable trajectories if common cycles exist in their trajectory. This limitation highlights the main issue in looking at probability distributions alone: temporal information is not captured.

To address the absence of local temporal information, transition matrices are constructed for each patient using the frequency of observed transitions. Namely, a transition matrix T has entries T (n)i,j corresponding to the proportion of transitions from action symbol i → j. Transition matrices for each patient are shown in eMethods Figures 12 and 13. Patients 9, 10, 12 and 19 all share a characteristic transition along the main diagonal corresponding to Nursing Intervention → Nursing Intervention. The main diagonal transition exists due to the high proportion of Nursing Intervention, as frequently occurring action symbols are more probable to transition to themselves. Patients 11 and 13 also exhibit this characteristic transition along the main diagonal due to their high proportion of a singular action symbol. Across all trajectories, only five transitions were unaccounted for, indicating that almost all transitions are possible and present in this dataset. The Assessment → Medical Intervention transition occurred in all patients except for Patient 16. Similarly, Assessment → Assessment and Medical Intervention → Assessment occurred in all but two patients.

© 2026 Milroy H et al. *JAMA Network Open*.

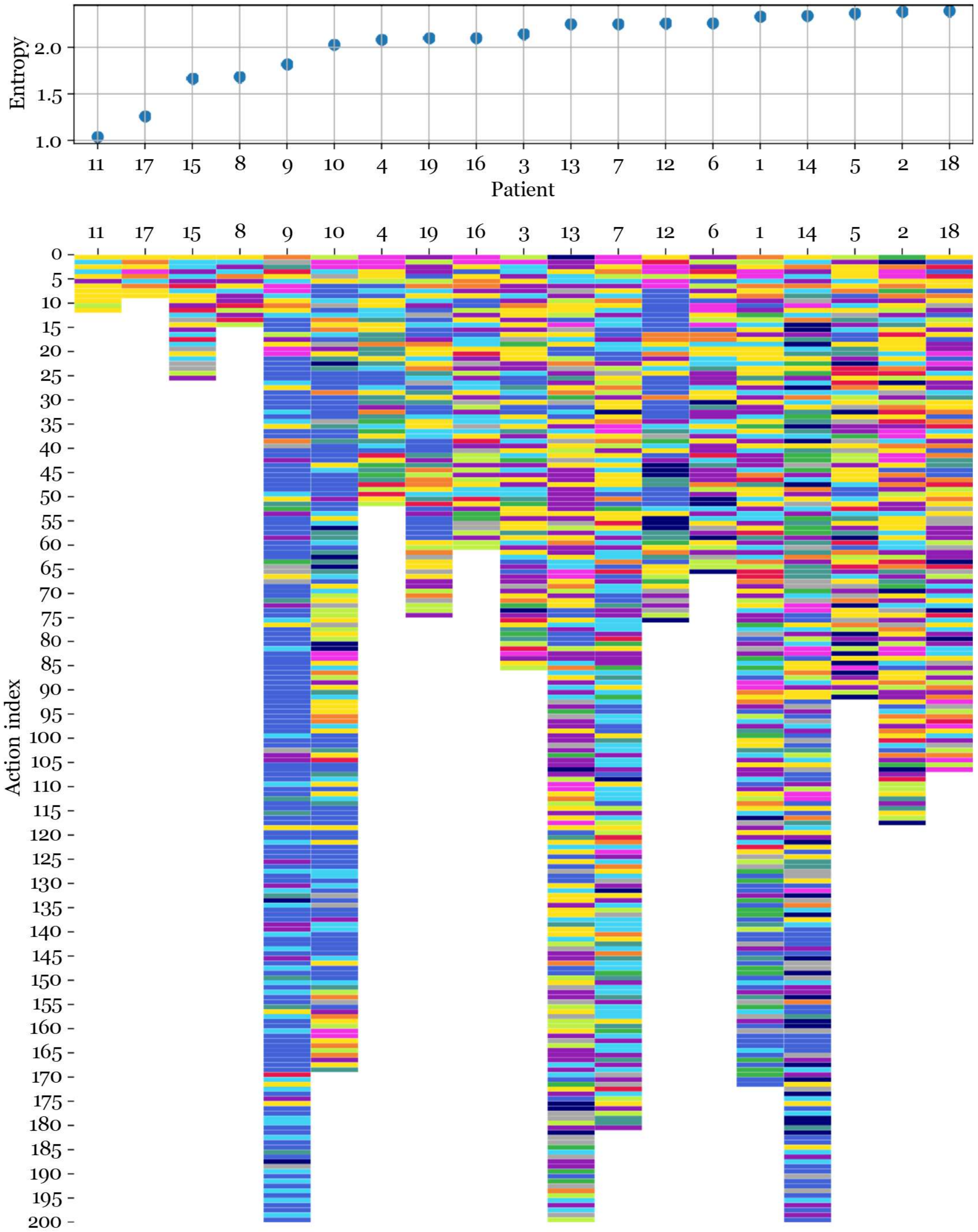

eMethods Figure 11. Patient-specific entropy and symbolic sequence trajectories. (Top) Shannon entropy of action-symbol probability distributions calculated for each patient's symbolic sequence. Lower entropy values indicate highly skewed sequences dominated by a small number of actions, whereas higher entropy values correspond to more balanced distributions across action types. Entropy measures the variability of observed actions in a manner that is independent of the length of the symbol sequence (Bottom). Visual representation of symbolic sequences for all 19 patients, where each vertical line denotes an event coded into its respective action symbol. Sequences are ordered from left to right by increasing entropy to illustrate the spectrum of predictability across patients, ranging from trajectories dominated by single types (e.g., repeated nursing interventions) to those with diverse and complex patterns of care actions.

© 2026 Milroy H et al. *JAMA Network Open*.

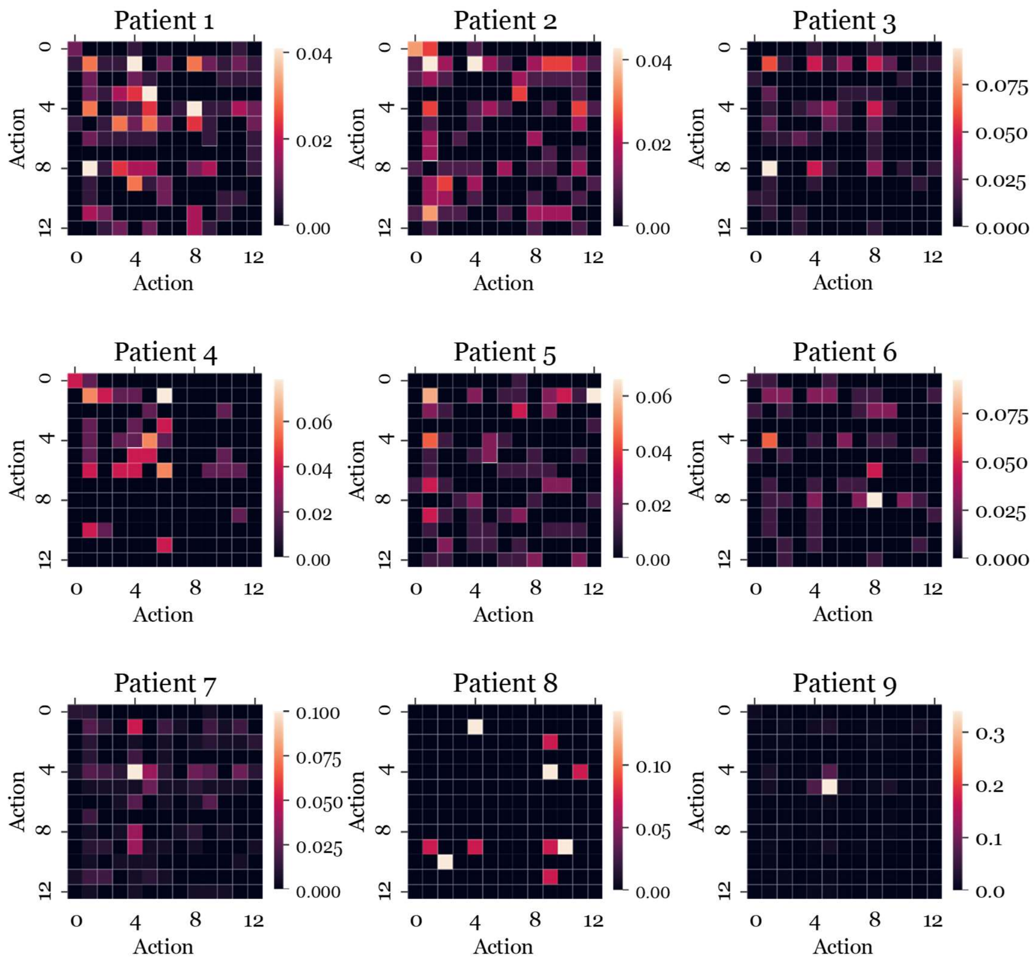

eMethods Figure 12. Transition matrices of Patients 1 to 9. Each heatmap represents the patient-specific transition matrix $T(n)$, where entry $T_{i,j}$ corresponds to the relative frequency of transitions from action symbol i to action symbol j. Action symbols were derived by coarse-graining interaction and outcome data into thirteen categories (Presentation, Assessment, Disposition, Intervention - AMHW, Medical, Nursing, Other, Liaison - External, Family, Internal, Referral - External, Internal, and Response). Brighter intensities indicate higher transition probabilities. Patterns show considerable heterogeneity across patients. For example, Patient 9's trajectory is dominated by self-transitions along the Nursing Intervention diagonal, reflecting repeated nursing actions, whereas Patients 1 and 7 demonstrate more distributed transitions across assessment, intervention, and disposition categories. The presence and frequency of these local temporal action sequences provide a basis for clustering patients and identifying characteristic clinical pathways.

© 2026 Milroy H et al. *JAMA Network Open*.

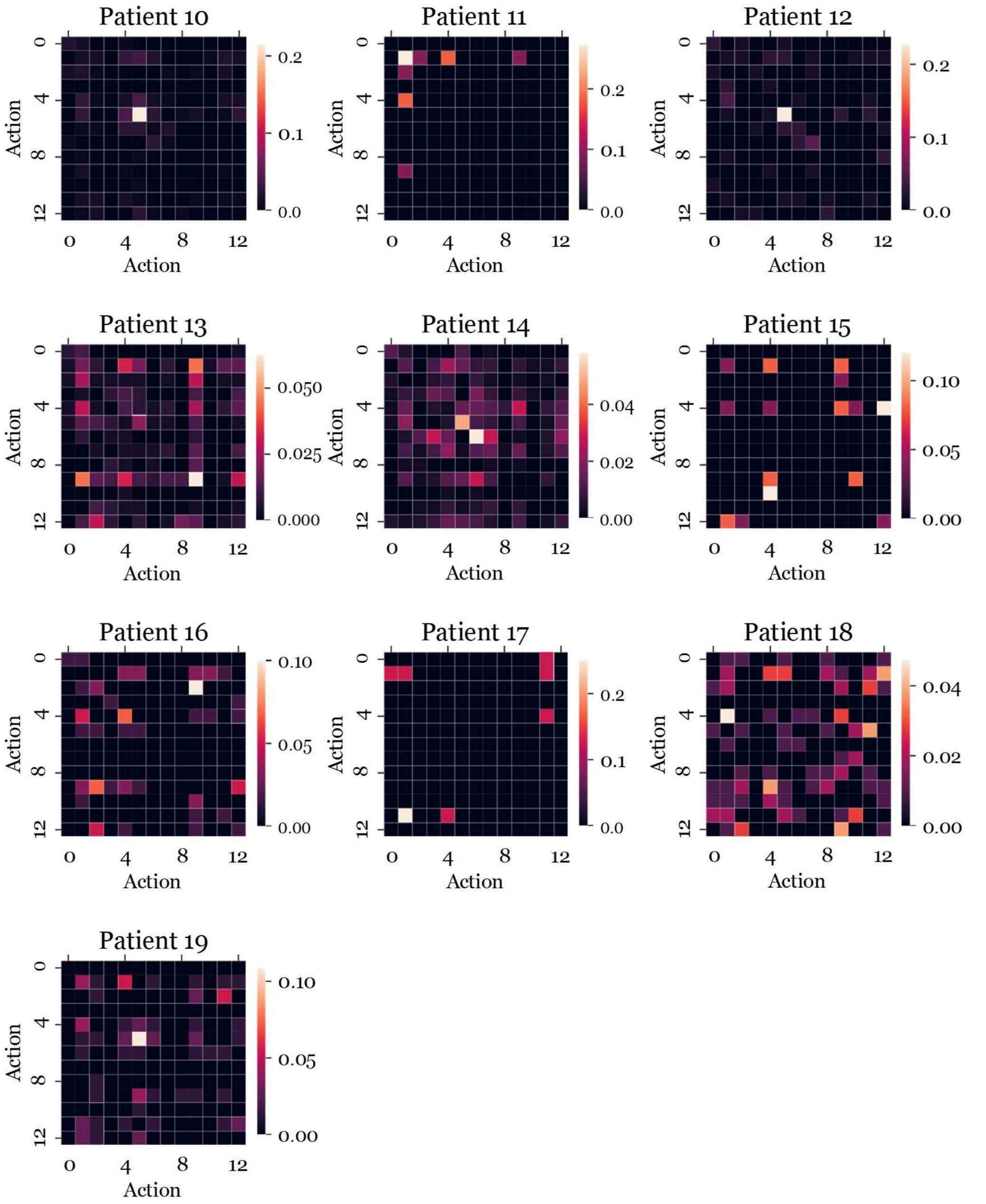

eMethods Figure 13. Transition matrices of Patients 10 to 19. Patient-specific transition matrices *T*(*n*) are shown, where entry *Ti,j* represents the proportion of observed transitions from action symbol *i* to action symbol *j*. Action symbols (Presentation, Assessment, Disposition, Interventions - AMHW, Medical, Nursing, Other, Liaison - External, Family, Internal, Referrals - External, Internal, and Response) were derived by coarse-graining observed interaction and outcome events. Brighter intensities denote higher transition probabilities. Several patients, including Patients 10, 12 and 19, exhibit strong self-transitions along the Nursing Intervention diagonal, reflecting repeated cycles of nursing actions, while Patient 13 shows a similar concentration linked to a dominant action category. In contrast, patients with broader matrices (e.g., 14 and 18) demonstrate more diverse transitions across assessments, dispositions, and referrals, indicative of complex or cyclical care pathways.

© 2026 Milroy H et al. *JAMA Network Open*.

We construct A distance matrix D by taking pairwise Jaccard distances between transition matrices. Namely, Dn,m = dJ (T (n), T (m)) for n, m = 1, 2, . . . , N = 19. Choice of ε is made by varying ε between Dmin and Dmax. As ε is increased, the network becomes more densely connected until the network is complete and forms one whole community, thus maximizing modularity. Thus, we cannot choose ε to maximize modularity. Instead, we increase ε only until an appropriate number of communities can be distinguished. Further increasing ε will only increase the density of edges, allowing for less discrimination between unique topological connectivities. eMethods Figure 14 shows that at ε ≈ 0.55, increasing ε causes singleton nodes to start forming detectable communities. At ε ≈ 0.8, community detectability stops increasing rapidly. Thus, we choose ε = 0.8 as our threshold. The resulting communities are shown in eMethods Figure 15.

Patients 8, 15, 11 and 17 correspond to the four symbolic sequences with least length. Therefore, it is no surprise that these four patients were grouped singularly or with one other node. As stated previously, the short symbolic sequences do not necessitate enough symbols for a fully populated transition matrix, making them further away from every other patient. For the remaining three clusters, we examine presentation to determine any correlation.

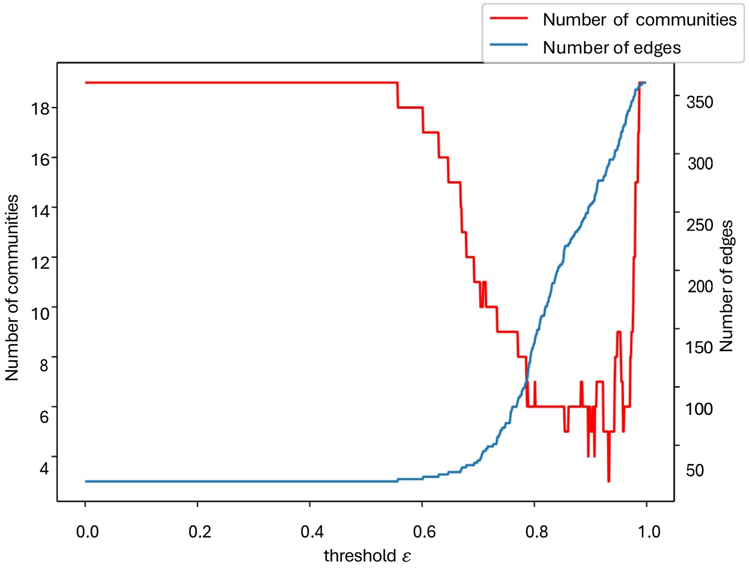

eMethods Figure 14. Distance threshold (ε) against number of communities and number of edges.
The figure shows the effect of varying the community detection threshold parameter ε on the connectivity and clustering of symbolic patient trajectories. The x-axis represents the distance threshold ε used in the ε-nearest neighbors construction, while the left y-axis (blue line) shows the number of detected communities and the right y-axis (orange line) shows the total number of edges in the constructed network. As ε increases from the minimum pairwise Jaccard distance between transition matrices (Dmin) to the maximum (Dmax), the number of communities decreases, and the network becomes progressively denser. At low ε, patients are isolated into many small or singleton communities due to sparse connectivity. Around ε ≈ 0.55, patients begin to aggregate into larger communities, and by ε ≈ 0.8 the number of new communities stabilizes while edges continue to accumulate. On this basis, ε = 0.8 was selected as an appropriate threshold for community detection, as it maximizes cluster interpretability without collapsing the network into a single undifferentiated community.

© 2026 Milroy H et al. *JAMA Network Open*.

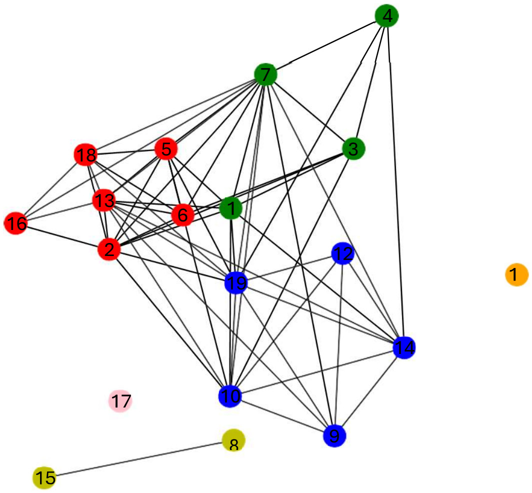

eMethods Figure 15. Detected communities of symbolic sequences using ε-nearest neighbour clustering (ε = 0.8). Each node represents an individual Aboriginal patient's symbolic sequence, constructed from observed clinical interactions and outcomes. Pairwise distances between patients were computed using Jaccard similarity on their transition matrices, and a network was formed by linking patients whose distance fell below the ε threshold (ε = 0.8). Community detection with modularity maximisation identified six clusters, visualised here by node colour. Patients within the blue, green, and red clusters demonstrate stronger internal similarity in their transition structures than between-cluster connections, suggesting shared patterns in disposition and referral transitions within these groups. Smaller singleton or two-patient clusters (green, pink, orange) typically correspond to patients with very short symbolic sequences, limiting the number of observable transitions. These patterns suggest that while overall separation between clusters was limited, patients within each group shared more closely aligned clinical trajectories and disposition behaviours than with patients in other clusters.

Modes of presentation are identified from the Presentation column of the dataset and shown in eMethods Table 3. Of the three remaining clusters of size 2 or greater, only the blue cluster has highly associated presentation types, with 3 of 5 presenting by themselves and 3 of 5 presenting with a police officer or ambulance. However, the red and green clusters contain a spectrum of presentations with no clear correlation. Thus, the clusters identified cannot be solely attributed to the presentation of each patient, and the presentation of a patient cannot be solely attributed to these clusters. We aim to quantify some of the structural features that are characteristic to these clusters.

eMethods Table 3. Patient presentation modes. Modes of presentation to the GSMHS for each participating Aboriginal patient (N = 19). Presentations were classified into four main categories: (i) Self – the patient presented independently; (ii) Associate – the patient was accompanied by family members, friends, or Aboriginal Mental Health Workers; (iii) External Referral – the patient was referred or transferred from another health service (e.g., GP, Great Southern Aboriginal Health Service (GSAHS), Royal Flying Doctor Service (RFDS), or other mental health services); and (iv) Police and Ambulance – the patient was brought in by emergency services. Several patients had multiple presentation pathways across different episodes, and categories are listed as reported in the event log data.
Abbreviations: GSAHS = Great Southern Aboriginal Health Service; RFDS = Royal Flying Doctor Service; GP = General Practitioner; OCCP = Occupational Physician; TFMHS = Transferred/referred from mental health service.

Patient	Self	Associate	External Referral	Police and Ambulance
1	Self	AMHW, Family		Police
2	Self	AMHW	GSAHS	
3				Transport officer
4			RFDS	
5			GSMHS, PALM	
6	Self	Family		Ambulance, Police
7	Self	Family	GP	
8			GP, OCCP	
9	Self	Family	GP	Police
10	Self			Transport officer
11			GP	
12				Transport officer
13	Self,	AMHW, Family	GSAHS, GP TFMHS	Police
14	Self		TFMHS	Ambulance, Police
15			GP	
16	Self			
17				Transport officer
18			GP	Ambulance
19			TFMHS	

© 2026 Milroy H et al. *JAMA Network Open*.

eAppendix. Qualitative Data analysis

De-identified transcripts were analysed using reflexive thematic analysis as described by Braun and Clarke [13], and coded collaboratively in NVivo. Analysis proceeded through familiarization with the transcripts, generation of initial codes, iterative grouping of codes into broader categories and candidate themes, review and refinement of themes across the dataset, definition and naming of themes and subthemes, and final write-up. Coding was conducted primarily using an inductive approach grounded in participants' accounts, with the Aboriginal SEWB framework [14] used as a sensitizing lens to refine interpretation and ensure that culturally relevant dimensions were not overlooked. Initial coding was led by BCB in collaboration with MC, MT, and TP, and an iteratively refined codebook was developed during analysis. Differences in coding and theme development were resolved through reflective team discussion and refinement of code and theme definitions, consistent with reflexive thematic analysis, rather than through interrater reliability testing. Aboriginal investigators contributed cultural interpretation and validation throughout theme development. To support rigor and credibility, preliminary findings were reviewed with community partners and the Aboriginal Reference Group, and qualitative interview participants were invited to comment on the interpretation of findings as part of participant feedback on interpretations. Feedback was received from two participants, both of whom expressed support for the interpretation and did not suggest changes to the themes or subthemes.

© 2026 Milroy H et al. *JAMA Network Open*.

eTable 1. Demographic and clinical characteristics of study participants in quantitative (n=19) and qualitative (n=7) analyses. The figure displays distributions for mean age (±SD), gender (n and %), and primary psychiatric diagnoses (n and %). Participants may have multiple diagnoses; therefore, percentages within diagnosis categories may total more than 100%.

**Characteristic**	**Qualitative** (n=7)	**Quantitative** (n=19)
Age, years (mean ±SD)	44.0 ± 17.8	38.4 ± 15.9
Male, n (%)	3 (42.9)	9 (47.4)
Female, n (%)	4 (57.1)	10 (52.6)
**Diagnosis, n (%)**		
Psychotic Disorder	2 (33.3)	6 (31.6)
Mood Disorder	3 (42.9)	8 (42.1)
Anxiety Disorder	1 (14.3)	2 (10.5)
Organic Disorder	0 (0.0)	1 (5.3)
Personality Disorder	2 (28.6)	6 (31.6)
Psychosocial Problem	3 (42.9)	12 (63.1)
Alcohol and Drug Problem	3 (42.9)	11 (57.9)
Stress/Trauma Problem	3 (42.9)	5 (26.3)

© 2026 Milroy H et al. *JAMA Network Open*.

eTable 2: Reporting of the mixed-methods study in relation to COREQ and GRAMMS criteria.

COREQ = Consolidated Criteria for Reporting Qualitative Research; GRAMMS = Good Reporting of a Mixed Methods Study

Domain/framework	Key reporting domain	How addressed in this study
COREQ	Research team and reflexivity	Qualitative interviews were facilitated by 2 Aboriginal researchers using culturally grounded research yarning. Aboriginal investigators contributed to cultural interpretation throughout analysis, and coding and theme development were refined through reflective team discussion.
COREQ	Interviewer positioning	The qualitative component was grounded in Indigenous methodology and Aboriginal leadership through the Aboriginal Participatory Action Research framework and culturally appropriate yarning methods.
COREQ	Study design	A qualitative component was embedded within a convergent mixed-methods design examining Aboriginal mental health care pathways at the Great Southern Mental Health Service in Western Australia.
COREQ	Participant selection	Aboriginal adults who had accessed the service in the preceding 12 months were recruited. Seven participated in qualitative interviews, and 6 contributed to both qualitative and quantitative components.
COREQ	Setting	Interviews explored experiences of psychiatric and community mental health care in a regional public mental health service in Western Australia.
COREQ	Ethical considerations and consent	Ethical approval was granted by the Western Australian Aboriginal Health Ethics Committee and the relevant research governance body. All participants provided informed consent.
COREQ	Data collection method	Data were collected through research yarning, an Indigenous qualitative method using culturally grounded, relational, participant-led conversation. Interviews were audio-recorded, transcribed, and de-identified.
COREQ	Interview guide	The yarning guide was co-developed with the Aboriginal governance team to explore experiences of cultural safety in mental health services.
COREQ	Participant safety	Direct questioning about trauma or clinical details was avoided unless raised by participants, and participant wellbeing was prioritised throughout data collection.
COREQ	Data analysis	Transcripts were analysed using reflexive thematic analysis. Analysis included familiarisation, coding, development of candidate themes, review and refinement of themes, and final naming and write-up.

© 2026 Milroy H et al. *JAMA Network Open*.

COREQ	Coding process	Initial coding was led by BCB in collaboration with MC, MT, and TP, with iterative refinement of a codebook through team discussion.
COREQ	Theoretical orientation	Coding was primarily inductive and grounded in participants' accounts, while the Aboriginal Social and Emotional Wellbeing framework informed interpretive refinement as a sensitising lens.
COREQ	Techniques to enhance trustworthiness	Preliminary findings were reviewed with community partners and the Aboriginal Reference Group, and qualitative participants were invited to comment on interpretations. Feedback was received from 2 participants, who supported the interpretation and did not suggest changes to themes or subthemes.
COREQ	Presentation of findings	Thematic findings are presented as one overarching theme with 3 interconnected subthemes, with participant quotations provided in the eTable 5
GRAMMS	Justification for mixed methods	Mixed methods were used to examine Aboriginal mental health care pathways through both lived experience and quantitative system analysis, enabling examination of structural dependencies, information flow, and cultural safety experiences.
GRAMMS	Mixed-methods design	The study used a convergent mixed-methods design with qualitative and quantitative components given equal weight.
GRAMMS	Description of each method	The quantitative component included network analysis and trajectory clustering based on 1108 documented clinical interactions from 19 patients. The qualitative component included in-depth yarning interviews with 7 Aboriginal adults analysed using reflexive thematic analysis.
GRAMMS	Sampling and participants	Twenty Aboriginal adults were recruited overall; 19 contributed quantitative data, 7 participated in interviews, and 6 contributed to both components.
GRAMMS	Integration	After independent analyses, findings were integrated using a merging approach through side-by-side comparison in a joint display to support integrated interpretation and generate meta-inferences.
GRAMMS	Interpretation of integrated findings	Joint sense-making sessions involving Aboriginal and quantitative researchers informed interpretation of the integrated findings.
GRAMMS	Outputs of integration	The joint display was used to generate meta-inferences regarding the structural centrality but inconsistent availability of Aboriginal Mental Health Workers, fragmented care pathways, patient burden as intermediaries, and the need for culturally safe system redesign.

© 2026 Milroy H et al. *JAMA Network Open*.

GRAMMS	Insights gained from mixing	Integration showed how quantitative network structure and trajectory patterns could be interpreted alongside lived experiences of cultural safety, kinship, trauma, and service fragmentation, producing insights not available from either component alone.
GRAMMS	Limitations of integration	Because only 6 participants contributed to both components, integration was conducted primarily at the level of patterns across datasets rather than through full participant-level matching.

© 2026 Milroy H et al. *JAMA Network Open*.

eTable 3. Quality Appraisal Tool for Aboriginal and Torres Strait Islander Studies: Evaluation of Our Study's Criteria Fulfillment

**Question**	**Answer** (Yes/Partially/No/Unclear)
Did the research respond to a need or priority determined by the community?	**Yes**<br>• This research aims to contribute to improving mental health care for Aboriginal and Torres Strait Islander peoples, addressing a community priority identified through close consultation with Aboriginal community partners (Greater Southern Aboriginal Health Planning Forum and an Aboriginal Reference Group) and researchers.<br>• The need for culturally safe mental health care was re-affirmed through the co-design of our broader research program [15].
Was community consultation and engagement appropriately inclusive?	**Yes**<br>• This research was conducted in close collaboration with Aboriginal community partners and researchers, both before the commencement of the project and continuously throughout its implementation.<br>• An iterative co-design and consultation process was carried out with local Aboriginal organizations, including the Great Southern Aboriginal Health Planning Forum and an Aboriginal Reference Group.
Did the research have Aboriginal and Torres Strait Islander research leadership?	**Yes**<br>• This research is led by Aboriginal and Torres Strait Islander researchers, with an Aboriginal executive team and Aboriginal lead investigators.
Did the research have Aboriginal and Torres Strait Islander governance?	**Yes**<br>• This research ensured that Indigenous governance and decision-making were central to the management and interpretation of the data, guided by an Aboriginal-led research team and Aboriginal research partners, with oversight from an Aboriginal Reference Group.
Were local community protocols respected and followed?	**Yes**<br>• By following consultations with local Aboriginal organizations<br>• Interviews employed yarning as a method of data collection.<br>• Gender considerations were adhered to through the employment of a female and male worker who led the yarning.<br>• Interviewers received specialized training in culturally appropriate engagement and reflexivity.<br>• Non-Indigenous participants involved in the research benefited from mentoring and training, including the completion of the Dance of Life program by Professor Helen Milroy [16] and participation in the Social and Emotional Wellbeing Gathering.<br>• Recruitment of participants was conducted in close collaboration with the treating team and local AMHWs who additionally offered advice on communicating with potential participants in a way that aligned with cultural safety practices.<br>• Participants could choose their interview location, including the Great Southern Mental Health Service, the Rural Clinical School in Albany, or the Albany public library
Did the researchers negotiate agreements in regard to rights of access to Aboriginal and Torres Strait Islander peoples' existing intellectual and cultural property?	**Yes**<br>• Aboriginal people have ownership over the data, demonstrated through the Aboriginal leadership team.

© 2026 Milroy H et al. *JAMA Network Open*.

Did the researchers negotiate agreements to protect Aboriginal and Torres Strait Islander peoples' ownership of intellectual and cultural property created through the research?	**Yes**<br>• This research was conducted within an Indigenous knowledge framework, empowering Aboriginal and Torres Strait Islander peoples to preserve, manage, safeguard, and advance the knowledge generated from this research.
Did Aboriginal and Torres Strait Islander peoples and communities have control over the collection and management of research materials?	**Yes**<br>• Through the Aboriginal Participatory Action Research (APAR) approach.<br>• Participants provided written informed consent and were aware of their right to withdraw at any time without consequences.<br>• The researchers implemented rigorous data security protocols to protect participants' identities and maintain confidentiality, with all data aggregated and de-identified.<br>• Findings were first shared with the Aboriginal research team and community partners for review and feedback prior to any external dissemination.<br>• A feedback loop was offered to participants, allowing them the opportunity to review and provide input on the interpretation of the data.
Was the research guided by an Indigenous research paradigm?	**Yes**<br>• This research employed APAR and integrated Aboriginal perspectives and values throughout the study.
Does the research take a strengths-based approach, acknowledging and moving beyond practices that have harmed Aboriginal and Torres Strait peoples in the past?	**Yes**<br>• The project aims to improve cultural safety in mental health services, by privileging the voices and knowledges held by Aboriginal peoples.<br>• Provisions were made for referrals to appropriate support agencies in case of participant distress during interviews.
Did the researchers plan and translate the findings into sustainable changes in policy and/or practice?	**Yes**<br>• The results from this project aim to inform a Cultural Safety Framework.
Did the research benefit the participants and Aboriginal and Torres Strait Islander communities?	**Yes**<br>• The results from this research aim to improve cultural safety of mental health services. It is possible that the local service will adopt recommendations based on the findings of this paper.
Did the research demonstrate capacity strengthening for Aboriginal and Torres Strait Islander individuals?	**Yes**<br>• Two Aboriginal people were employed to lead the yarning interviews and were invited as authors on this paper.
Did everyone involved in the research have opportunities to learn from each other?	**Yes**<br>• Continuous collaboration, consultation, and training between researchers and Aboriginal partners/community members facilitated opportunities for mutual two-way learning.

eTable 4. Yarning guide for the qualitative component of the study. The questions listed were developed as a draft and were iteratively refined through community consultation, following a participatory qualitative approach. This iterative process ensured that the yarning guide remained responsive to community feedback and was adapted throughout the research.

Questions
• Can you tell me a little about how you were referred to the great southern mental health service?<br>o Probe: who was the referral made by?<br>o Follow up question: is this the first mental health service you have been referred to?
• Once you were referred, what sort of contact did you have with the service?<br>o Probe: how frequent was the contact?<br>o Probe: how did they contact you or your family?<br>o Follow up question: what were your experiences of this initial contact?
• Have you been to an appointment yet?<br>o probe: do you have plans to attend an appointment?<br>o Follow up question: what are the reasons you have not attended an appointment?
• Is there anything that could be changed to help you attend an appointment?
• Who have been your main supports in your mental health care? (if they have been to an appointment)
• What were your feelings of going to speak with a psychologist/psychiatrist?
• What have your experiences been like with the staff at the hospital?
• Do you feel this service understands your culture?<br>o Probe: what does the service do or not do to show you this?<br>o Probe: have you had access to an Aboriginal mental health worker?
• Is there anything you would like to see improved about the service?
• Do you feel mental health services understand Aboriginal people and their culture?<br>o Probe: can you give me an example?<br>o Probe: can you describe what happened?
• How would you like culture to be recognised through mental health services?<br>o Probe: what do services do well for cultural safety<br>o probe: what do services do badly?
• What are some of the cultural principles that services need to be respectful of?<br>o (question for family members and carers): Are there additional cultural considerations for families and carers in mental health services?
• How can mental health services build cultural safety?<br>o Prompt: things that promote, enhance and empower consumer and clinicians' culture

© 2026 Milroy H et al. *JAMA Network Open*.

eTable 5. Participant quotes illustrating key themes from qualitative interviews. Selected participant quotations are presented, exemplifying key themes from qualitative interviews exploring Aboriginal peoples' experiences accessing the GSMHS in Kinjarling (Albany). Participant identifiers are provided to ensure authenticity while maintaining anonymity.

The Central Role of Culture in Mental Health and Wellbeing

- 'And my history, what people don't understand is that as Aboriginal people, we've got spiritual, cultural and then there's mental health. I've been through the three of them. Spiritual is my everyday life. Cultural is my everyday life because I'm Aboriginal. Mental health is affected when people don't believe me that what I'm going through is cultural or spiritual.' (P16, female)
- 'I believe it's my journey that I've asked my ancestors to take me on to heal. And it was a disrespect to my ancestors for doing it in the first place. And like as blackfellas we never had tobacco, drink, drugs, whatever. But then we didn't have all this other society stuff either, too. And that's not saying that – we just got to get back to basics where we feel really good. And back out on country, doing things on country, yeah.' (P7, female)
- 'I had a mental breakdown, and I was scared about opening up to the doctors and stuff down here [in Albany] because I've had a bad experience. They listened to everything that I had to say. They didn't judge me. They didn't say to me... They didn't say that my feelings weren't valid. They didn't say to me that anything that I've told them weren't valid. They listened to me. They didn't judge me because down here, they have more of understanding of Aboriginal people than Perth. That's what it comes down to.' (P16, female) 'There needs to be more Aboriginal people working in mental health. You need to be direct about how Aboriginal people don't give eye contact. […] And if you don't give eye contact, it doesn't mean that it's bad body language. They need to have cultural awareness. If there's no Aboriginal workers, there needs to be cultural awareness that we find eye contact intimidating.' (P16, female)
- 'And I don't believe that there should be women and men in the same ward. There should be a woman's section and a man's section.

  [Interviewer] […] So you're saying not mixed? […]
  No, not mixed, because we go to sleep in the same corridor as well as men's. That's against our culture.' (P2, female)
- 'I know there's one thing that I don't like there, is sometimes when they come and check on you during the night, they look through that little hole and they point the torch at you. Now, they point that torch straight in your eyes. And we know as blackfellas, what can happen too this light comes straight in your eyes, wakes you up, and the spirit comes through, right. So that happening is really quite disturbing. Not only does it wake you up, but the belief and the cultural stuff that we know, that happens.' (P7, female)
- 'Because blackfella way, maybe sometimes we're not meant to talk about our private lives. We don't say what's going on at home because a lot of time blackfellas be like, "Now don't go telling these wadjellas [Noongar word for whitefellas] what we're talking about. You don't tell no one our business.' (P16, female)

'The kinship stuff'

- 'And I said, "Well, actually yes, because yeah, I'd like to be able to talk about some deeper stuff here that I really can't talk with people who aren't getting it." […] Yeah, because there's a whole thing there about, yeah, our Indigenous families that… some people just don't get. Like the size of them and how many funerals we have to go to.' (P14, female)
- 'Yeah, I think I had a little thing when I was about in my 20s when my Nan passed, it was only short […] then all the way up to 2020 when I started taking the tablets that I'm on now because I, it kind of hit me pretty hard but it was the fallout plus the anxiety and just everything that I suppose or going way back to when I was a young fellow because that's where I did my mental health. I suppose with counselling and stuff has worked all that stuff out that it was triggers from way back when I was a kid that carried all the way through.' (P8, male)
- 'But it's not good at the moment because my kids aren't with me at this time. DCP have taken my baby and it's a 12-month order. So now I don't have any of my kids with me and I'm just an emotional wreck.' (P16, female)
- 'He doesn't have nothing to do with it. He does not condone us on drugs. So, I'm going to fix myself

© 2026 Milroy H et al. *JAMA Network Open*.

before I get acknowledged by my father.' (P2, female)

- 'I knew something had to change in my life. I knew I had to do something. I've only just started having these psychotic episodes. And I didn't fight my whole life to have my beautiful girls and to get a house and everything, from everything that I've been through, to end up a vegetable. I didn't. And I won't. And […] knowing that my children were going to be looked after by my aunty, who they respect, and who is a lovely lady, that made all the difference for me to be able to sit in here [inpatient at mental health] and relax – or not relax, but sit in here and spend time and get things moving to make it all better, for me to get better, sorry.' (P7, female)
- 'And if I do talk about some of the trauma, and they start saying things like, "Cut off your family," and I'm like, "all right, there are some cultural problems here, we can't talk anymore. Thank you anyway." Because that's a different mentality. Granted a valid option, not one I have chosen, and it is a cultural thing because my family, we have this… you know, there's reasons why we're all like we are, there's a reason why things happened as they happened. […] All stemming from the research of colonisation, right? […] So, cutting them off is not something I want to do. Boundaries, good, moving to a different town, also good.' (P14, female)

Trauma and stress

- 'And the only way I was engaged to mental health is because my parents brought me in when I first had a nervous breakdown, when my child was moved out of my care and after the tragedy, death of my [family member] being [killed] down here.' (P2, female)
- 'I knew I was struggling but also too there was just the signs were popping up like oh I suppose not suicidal thoughts but just thoughts of had enough and just wanted to, didn't want to harm myself but just couldn't kind of cope there' (P8, male)
- '[…] if I was brought here in an ambulance, or the back of a police truck or whatever, if that AMHW was there straightaway, then I would feel safer. Because at the end of the day, I mean, ambulances have beautiful people, but at the end of the day, policemen frighten me. What they will produce, or how they were formed to eradicate half castes[1], right from the word go, is not nice. If you come into the back of the thing, if AMHWs were right there, then it feels safer. They would be able to calm us a bit more, have more of an understanding of what's been going on with us.' (P7, female)

  [1]The term half-caste was historically used in Australia to describe individuals of mixed Aboriginal and non-Indigenous heritage. is now accepted as an offensive term.

- 'Because the spaces that we want don't exist yet. This place where you can come and just go, "I don't have to explain my whole culture to you. I don't have to explain anything."'(P14, female)

eTable 6: Characteristics of Culturally Safe Mental Health Services and Proposed Strategies for Implementation in a Community Mental Health Service and Inpatient Unit
Aboriginal Mental Health Workers (AMHWs); Emergency Department (ED).

Characteristics	Proposed Strategies for Implementation	
	Community Mental Health Service	Inpatient Unit
**People who listen, understand and do not judge**	More AMHWs and involvement as early on as possible.<br>Ongoing cultural training for all staff aiming to deepen understanding and appreciation of Aboriginal ways of life moving beyond cultural 'awareness and knowledge' to 'acknowledgement' of Aboriginal experiences.<br>Working together with non-Aboriginal champions within the service.	Developing culturally safe self-referral protocols.<br>Involvement of AMHWs during ED presentations.<br>Cultural training for all ED staff.<br>Training on trauma-informed care for all ED staff on the impact of Police involvement in involuntary admissions.<br>On-going cultural training for all ward staff aiming to deepen understanding and appreciation of Aboriginal ways of life moving beyond cultural 'awareness and knowledge' to 'acknowledgement' of Aboriginal experiences.<br>Working together with non-Aboriginal champions within the service.
**Trauma-informed care**		
**Culturally-safe environment**	Calm waiting room area allowing patients to have their own personal space.	Separate gender wards.<br>Cultural activities including cooking (e.g. damper) and Aboriginal art.<br>Promote healing by facilitating connection to the land, outside the boundaries of the hospital (e.g. walks to the beach, activities on Country)

© 2026 Milroy H et al. *JAMA Network Open*.

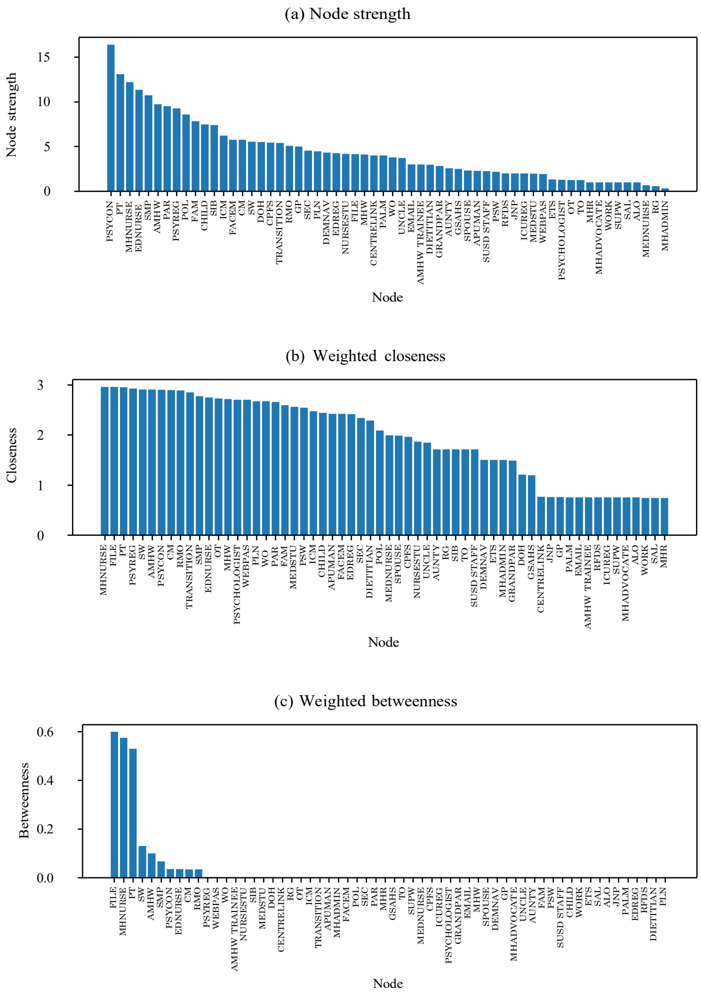

eFigure 1. Weighed centrality measures of the Clinical Interaction Network. Three metrics are shown for each node: (a) node strength, reflecting cumulative interaction frequency; (b) weighted closeness centrality, representing the inverse of average shortest path distances weighted by interaction frequency; and (c) weighted betweenness centrality, indicating the degree to which a node lies on the shortest weighted paths between other nodes. Key nodes such as the Mental Health Nurse, Patient File, and Aboriginal Mental Health Worker (AMHW) display high centrality across these metrics, highlighting their pivotal roles in care coordination and information flow within Aboriginal psychiatric service delivery at GSMHS.

eReferences

1. McCullough MH, Small M, Jayawardena B, Hood S. Mapping clinical interactions in an Australian tertiary hospital emergency department for patients presenting with risk of suicide or self-harm: Network modeling from observational data. *PLoS Med* 2024; **21**(1): e1004241.
2. Theiler J, Eubank S, Longtin A, Galdrikian B, Doyne Farmer J. Testing for nonlinearity in time series: the method of surrogate data. *Physica D: Nonlinear Phenomena* 1992; **58**(1): 77-94.
3. Lancaster G, Iatsenko D, Pidde A, Ticcinelli V, Stefanovska A. Surrogate data for hypothesis testing of physical systems. *Physics Reports* 2018; **748**: 1-60.
4. Erdös P, Rényi A. On the evolution of random graphs. The Structure and Dynamics of Networks. Princeton: Princeton University Press; 2006: 38-82.
5. Fosdick BK, Larremore DB, Nishimura J, Ugander J. Configuring Random Graph Models with Fixed Degree Sequences. *SIAM Review* 2018; **60**(2): 315-55.
6. Gkantsidis C, Mihail M, Zegura EW. The Markov Chain Simulation Method for Generating Connected Power Law Random Graphs. Workshop on Algorithm Engineering and Experimentation; 2003; 2003.
7. Newman M. Networks: Oxford University Press; 2018.
8. Nolan-Isles D, Macniven R, Hunter K, et al. Enablers and Barriers to Accessing Healthcare Services for Aboriginal People in New South Wales, Australia. *Int J Environ Res Public Health* 2021; **18**(6).
9. Cao R, Shang P. Similarity measurement of symbolic sequence based on complexity estimate and dynamic time warping. *Nonlinear Dynamics* 2024; **112**(21): 19055-70.
10. Faure P, Lesne A. RECURRENCE PLOTS FOR SYMBOLIC SEQUENCES. *International Journal of Bifurcation and Chaos* 2010; **20**(06): 1731-49.
11. Brida JG, Punzo LF. Symbolic time series analysis and dynamic regimes. *Structural Change and Economic Dynamics* 2003; **14**(2): 159-83.
12. Ferreira LN, Zhao L. Time series clustering via community detection in networks. *Information Sciences* 2016; **326**: 227-42.
13. Braun V, Clarke V. Using thematic analysis in psychology. *Qualitative research in psychology* 2006; **3**(2): 77-101.
14. Gee G, Dudgeon P, Schultz C, Hart A, Kerrie K. Aboriginal and Torres Strait Islander Social and Emotional Wellbeing. In: Dudgeon P, Milroy H, Walker R, eds. Working Together: Aboriginal and Torres Strait Islander Mental Health and Wellbeing Principles and Practice 2ed. Canberra: Commonwealth of Australia; 2014: 55-68.
15. Milroy H, Kashyap S, Collova J, et al. Co-designing research with Aboriginal and Torres Strait Islander consumers of mental health services, mental health workers, elders and cultural healers. *Australian Journal of Rural Health* 2022; **30**(6): 772-81.
16. Milroy H. The Dance of Life. 2006. https://www.ranzcp.org/clinical-guidelines-publications/in-focus-topics/aboriginal-and-torres-strait-islander-mental-health/the-dance-of-life.

© 2026 Milroy H et al. *JAMA Network Open*.
