# Supplementary material for: Mapping Aboriginal Mental Health Journeys Through Psychiatric Care Systems
Source: JAMA Netw Open. 2026 May 20;9(5):e2613904. doi: 10.1001/jamanetworkopen.2026.13904 (PMC13191385; doi:10.1001/jamanetworkopen.2026.13904)
Supplement: Supplement 2. — Data Sharing Statement [file jamanetwopen-e2613904-s002.pdf]

Data Sharing Statement

Milroy. Mapping Aboriginal Mental Health Journeys Through Psychiatric Care Systems. *JAMA Netw Open*. Published May 20, 2026. doi:10.1001/jamanetworkopen.2026.13904

Data
Data available: No

Additional Information
Explanation for why data not available: De-identified data were used in this study, protected under confidentiality provisions consistent with ethical guidelines for Aboriginal and Torres Strait Islander research. Analysis code/scripts are available from the corresponding author upon reasonable request.
